# Src-mediated PHB2 phosphorylation disrupts mitochondrial cristae through cardiolipin dissociation in hepatocellular carcinoma

**DOI:** 10.1016/j.redox.2026.104073

**Published:** 2026-02-07

**Authors:** Zhehua Shao, Xinnuo Yang, Binben Wang, Xuwen Wang, Duoduo Zhao, Bingchen Liu, Jinliang Nan

**Affiliations:** a Key Laboratory of Respiratory Disease of Zhejiang Province, Department of Respiratory and Critical Care Medicine, Second Affiliated Hospital of Zhejiang University School of Medicine, Hangzhou, Zhejiang, China; b Department of Cardiology of the Second Affiliated Hospital, School of Medicine, Zhejiang University, Hangzhou, China; c School of Mathematical Sciences, Zhejiang University, Hangzhou, China; d College of Information Science and Electronic Engineering, Zhejiang University, Hangzhou, China; e Bone Marrow Transplantation Center of the First Affiliated Hospital, Zhejiang University School of Medicine, Hangzhou, China; f State Key Laboratory of Transvascular Implantation Devices, Hangzhou, China; g Cardiovascular Key Laboratory of Zhejiang Province, Hangzhou, China; h Research Center for Life Science and Human Health, Binjiang Institute of Zhejiang University, Hangzhou, China

**Keywords:** Hepatocellular carcinoma (HCC), Mitochondrial cristae, Cardiolipin, Prohibitin, Src-mediated phosphorylation, Redox homeostasis

## Abstract

Hepatocellular carcinoma (HCC) displays mitochondrial dysfunction characterized by disrupted redox homeostasis and cristae disorganization, yet the underlying molecular mechanisms are unclear. We reveal that Src kinase phosphorylates prohibitin 2 (PHB2) at tyrosines Y34 and Y77 under oxidative stress, disrupting its interaction with cardiolipin and triggering PHB1/2 complex disassembly. This event activates the mitochondrial protease OMA1, promoting excessive cleavage of the cristae-shaping protein OPA1, leading to severe cristae remodeling. Consequent impairment of electron transport chain supercomplexes decreases NAD+/NADH ratio and complex I/II activities, creating conditions that promote enhanced electron leakage and oxidative stress. This mitochondrial dysfunction drives a metabolic shift from oxidative phosphorylation toward glycolysis, promoting tumor growth in xenograft models. Phosphomimetic PHB2 mutants (Y34E/Y77E) exacerbate these effects, whereas phosphorylation-resistant mutants (Y34F/Y77F) restore cristae integrity, normalize redox balance, and suppress tumor progression. Our findings establish Src-mediated PHB2 phosphorylation as a redox-sensitive molecular switch that drives HCC metabolic reprogramming by disrupting the PHB2-cardiolipin cristae axis. This phosphorylation event represents a targetable vulnerability for this malignancy with limited treatment options.

## Graphical abstract

**Figure:**
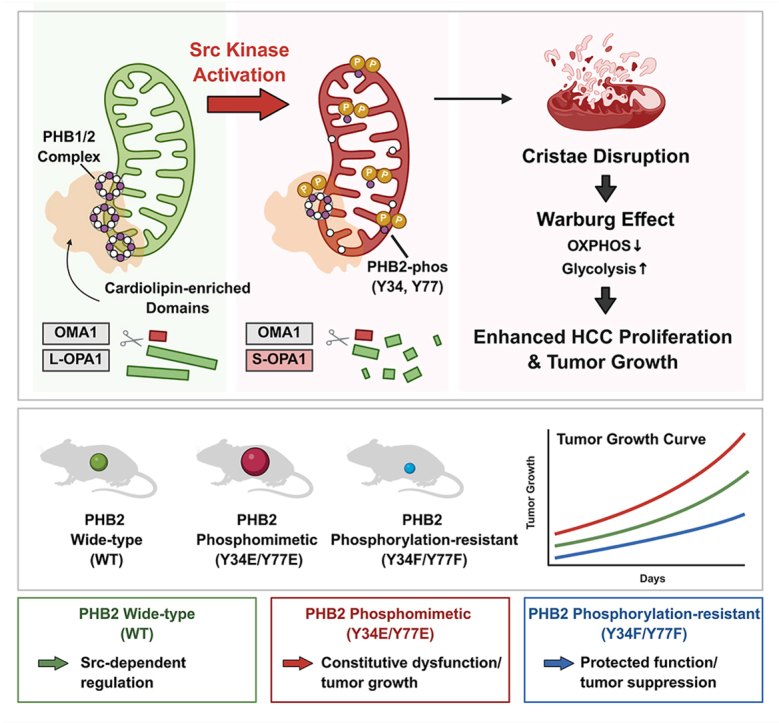

## 1 Introduction

Hepatocellular carcinoma (HCC) represents the third leading cause of cancer-related mortality worldwide, with over 900,000 new cases diagnosed annually [1]. Despite recent advances in systemic therapy, including the combination of atezolizumab plus bevacizumab, the median overall survival for advanced HCC remains disappointingly short at less than 20 months [2,3]. This limited therapeutic efficacy is largely attributed to metabolic plasticity, particularly the shift from oxidative phosphorylation (OXPHOS) to glycolysis, known as the Warburg effect [4,5].

This metabolic reprogramming fundamentally disrupts cellular redox homeostasis [6]. The reduced NAD^+^/NADH ratio in HCC cells reflects impaired electron transport and contributes to reductive stress, which can promote oxidative damage through compromised antioxidant defense and aberrant electron flow at respiratory complexes [7,8]. Src family kinases (SFKs), frequently activated in HCC, have emerged as critical regulators of this metabolic shift and may serve as redox-sensitive mediators. Recent evidence demonstrates that Src directly phosphorylates glycolytic enzymes, enhancing their activities and driving glycolytic flux, while elevated Src expression correlates with reduced OXPHOS complex expression and enhanced metastatic potential [[9], [10], [11]].

Beyond metabolic alterations, HCC cells display profound mitochondrial structural abnormalities directly impacting redox balance [12]. Transmission electron microscopy reveals that tumor mitochondria are significantly enlarged yet contain dramatically reduced cristae density, correlating with impaired respiratory complex activities, increased ROS production, and adverse clinical outcomes [13,14]. The prohibitin 1/2 (PHB1/2) complex forms ring-like structures on the inner mitochondrial membrane (IMM) and plays a crucial role in maintaining redox homeostasis by organizing respiratory supercomplexes and minimizing electron leakage [15]. Recent cryo-electron tomography revealed approximately 43 PHB1/2 complexes per crista, functioning as membrane scaffolds [16,17]. In addition, cardiolipin has been demonstrated to play crucial roles in maintaining cristae structural stability [18,19]. This mitochondria-specific phospholipid constitutes approximately 20% of IMM lipids and preferentially clusters at cristae junctions and tips, where it stabilizes respiratory supercomplexes and facilitates membrane curvature [20,21]. The interaction between cardiolipin and mitochondrial proteins is essential for cristae integrity, as disruption invariably leads to cristae disorganization and respiratory dysfunction [22]. While both PHB1/2 complexes and cardiolipin are indispensable for respiratory function, whether their interaction is required for cristae maintenance and how this relationship might be disrupted in cancer remains unknown.

The sensitivity of mitochondrial cristae structure to oxidative stress has been well documented, with reactive oxygen species capable of disrupting lipid-protein interactions and promoting proteolytic remodeling. Cardiolipin, highly enriched in polyunsaturated fatty acids, is particularly vulnerable to oxidative modification, and its peroxidation compromises cristae stability and respiratory chain organization [23]. Downstream of cristae structural changes, the OMA1-OPA1 proteolytic axis serves as a critical effector system for cristae remodelin. Under stress conditions, the zinc metalloprotease OMA1 cleaves the dynamin-like GTPase OPA1 from its long form (L-OPA1) to its short form (S-OPA1), with excessive cleavage resulting in cristae fragmentation and OXPHOS collapse [[24], [25], [26], [27]]. In HCC, aberrant OPA1 processing has been linked to enhanced cell migration and metabolic reprogramming [28]. Nevertheless, the upstream signals that trigger pathological OMA1 activation in liver cancer remain elusive.

In this study, we demonstrate that PHB2, but not PHB1, undergoes Src-mediated phosphorylation at tyrosine residues Y34 and Y77 specifically in HCC cells. This phosphorylation introduces negative charges that disrupt PHB2-cardiolipin interaction through electrostatic repulsion, triggering PHB1/2 complex disassembly, cytoplasmic PHB2 accumulation, and OMA1 activation. The resulting cristae disorganization drives metabolic shift from OXPHOS to glycolysis, promoting tumor growth. Our findings identify the Src-PHB2-cardiolipin axis as a critical regulatory mechanism for mitochondrial cristae organization and reveal PHB2 phosphorylation as a potential therapeutic target for restoring mitochondrial function in HCC.

## 2 Materials and methods

### 2.1 Cell culture and transfection

Human hepatocellular carcinoma cell line HepG2 and normal hepatocyte cell line LO2 were obtained from the Cell Bank of the Chinese Academy of Sciences (Shanghai, China) and authenticated by short tandem repeat (STR) profiling (HepG2: RRID:CVCL_0027; LO2: RRID:CVCL_6926). HEK293T cells were purchased from the American Type Culture Collection (ATCC CRL-3216; RRID:CVCL_0063). All cells were cultured in high-glucose DMEM (Gibco C11995500BT, 25 mM glucose, 4 mM glutamine, no pyruvate) additionally supplemented with 1 mM sodium pyruvate, 10% fetal bovine serum (FBS, Gibco) and 1% penicillin-streptomycin at 37 °C in a humidified atmosphere with 5% CO2. All cell lines tested negative for mycoplasma contamination using the MycoAlert Detection Kit (Lonza). HepG2 cells exhibited a doubling time of approximately 24 h, while LO2 cells showed a slower doubling time of approximately 36 h. For all experiments, cells were seeded and harvested at 70-80% confluence to ensure comparable metabolic states and avoid contact inhibition or nutrient depletion. Because of the different growth rates, the two cell lines were harvested based on confluence rather than at fixed time points. Transient transfections were performed using Lipofectamine 3000 (Invitrogen) according to the manufacturer's instructions. For stable cell line generation, cells were transduced with lentiviral vectors and selected with 2 μg/ml puromycin for 7 days.

### 2.2 Plasmid construction and site-directed mutagenesis

Human PHB2 cDNA was cloned into the pcDNA3.1-Flag vector using standard molecular cloning techniques. Point mutations (Y34F, Y77F, Y34E, Y77E, K6A, R17A, R54T, R71T) were introduced using the QuikChange II Site-Directed Mutagenesis Kit (Agilent Technologies) following the manufacturer's protocol. Human SRC cDNA was cloned into the pLVX-IRES-puro lentiviral vector for overexpression studies. All constructs were verified by Sanger sequencing to confirm the absence of unintended mutations.

### 2.3 RNA interference

Small interfering RNAs (siRNAs) targeting human SRC (5′-CACCTTTGTGGCCCTCTATGACT-3′), EGFR (5′-GAGGAAATATGTACTACGAAAAT-3′), INSR (5′-TCCACTATAACCCCAAACTCTGC-3′), and scrambled control siRNA were purchased from GenePharma (Shanghai, China). Cells were transfected with 50 nM siRNA using Lipofectamine RNAiMAX (Invitrogen) and analyzed 48-72 h post-transfection. As a negative control, a non-targeting scrambled siRNA pool (control-siRNA) was used at the same concentration. For stable knockdown, short hairpin RNA (shRNA) sequences were cloned into the pLKO.1-puro vector targeting human SRC (5′-CACCTTTGTGGCCCTCTATGACT-3′) and OMA1 (5′-CTGTATGGAATGATGCTTTTTCA-3′). Knockdown and overexpression efficiency were tested at RNA levels by qRT-PCR or protein levels by immunoblotting.

### 2.4 Western blotting and immunoprecipitation

Cells were lysed in RIPA buffer (50 mM Tris-HCl pH 7.4, 150 mM NaCl, 1% NP-40, 0.5% sodium deoxycholate, 0.1% SDS) supplemented with protease and phosphatase inhibitor cocktails (Roche). Protein concentrations were determined using the Pierce BCA Protein Assay Kit. For immunoprecipitation, 500 μg of protein lysate was incubated with 2 μg of primary antibody overnight at 4 °C with gentle rotation, followed by incubation with protein A/G agarose beads (Santa Cruz Biotechnology) for 2 h. Immunoprecipitates were washed three times with lysis buffer and eluted with SDS loading buffer. The following antibodies were used: *anti*-PHB2 (1:1000, Cell Signaling Technology #14085), *anti*-PHB1 (1:1000, Abcam ab75771), *anti*-phosphotyrosine (1:1000, Cell Signaling Technology #9411), *anti*-SRC (1:1000, Cell Signaling Technology #2109), *anti*-β-actin (1:5000, Sigma-Aldrich A5441), *anti*-HSP60 (1:1000, Cell Signaling Technology #12165), *anti*-OMA1 (1:1000, Santa Cruz Biotechnology sc-515788), *anti*-OPA1 (1:1000, BD Biosciences 612606), *anti*-VDAC (1:2000, Proteintech #66345‐1‐Ig), and *anti*-AFG3L2 (1:1000, Abcam ab139503).

### 2.5 Blue Native polyacrylamide gel electrophoresis (BN-PAGE)

Blue Native PAGE was performed as previously described [29]. Briefly, cells were resuspended in solubilization buffer (50 mM NaCl, 50 mM imidazole, 2 mM 6-aminohexanoic acid, 1 mM EDTA, pH 7.0) and solubilized with 1% digitonin for 15 min on ice. After centrifugation at 20,000 g for 30 min, supernatants were supplemented with Coomassie G-250 and loaded onto 3-12% gradient native gels. Electrophoresis was performed at 4 °C, starting at 100 V for 30 min, then 200 V until completion. Proteins were transferred to PVDF membranes (Millipore). After blocking by 5% (w/v) Non-Fat powered Milk-Tris-buffered saline tween (TBST) buffer, the protein was immunoblotted with primary antibodies at 4 °C overnight, washed and then incubated with the corresponding HRP-conjugated secondary antibody for 1 h. Signal was detected by chemiluminescence using ChemiDoc Touch Imaging System.

### 2.6 Subcellular fractionation

Mitochondrial and cytoplasmic fractions were isolated using the Mitochondria Isolation Kit (Thermo Scientific) according to the manufacturer's protocol. Briefly, cells were homogenized in isolation buffer using a Dounce homogenizer (20 strokes), followed by differential centrifugation at 750*g* for 10 min and 12,000 g for 15 min. The mitochondrial pellet was washed twice with isolation buffer. Fraction purity was validated by Western blotting using HSP60 as a mitochondrial marker and β-actin as a cytoplasmic marker.

### 2.7 Transmission electron microscopy (TEM)

Cells were fixed with 2.5% glutaraldehyde in 0.1 M phosphate buffer (pH 7.4) for 2 h, post-fixed with 1% osmium tetroxide for 1 h, dehydrated through graded ethanol series, and embedded in Spurr's resin. Ultrathin sections (70 nm) were cut using a Leica EM UC7 ultramicrotome, stained with uranyl acetate and lead citrate, and examined using a JEOL JEM-1400 transmission electron microscope at 80 kV. Images were captured using a Gatan CCD camera. Mitochondrial area and cristae density were quantified using ImageJ software by an investigator blinded to experimental conditions. For each condition, at least 100 mitochondria were analyzed from a minimum of 30 randomly selected cells across three independent experiments (approximately 30-40 mitochondria per experiment). Cristae density was calculated as the ratio of cristae membrane length to mitochondrial area.

### 2.8 High-resolution respirometry

Mitochondrial oxygen consumption was measured using the Oxygraph-2k system (OROBOROS Instruments, Innsbruck, Austria). The MiR05 buffer (Oroboros Instruments, Cat# 60101-01) contained 0.5 mM EGTA, 3 mM MgCl_2_·6H_2_O, 60 mM lactobionic acid, 20 mM taurine, 10 mM KH_2_PO_4_, 20 mM HEPES, 110 mM sucrose, and 1 g/L fatty acid-free BSA, pH 7.1.

**Fig. 1:**
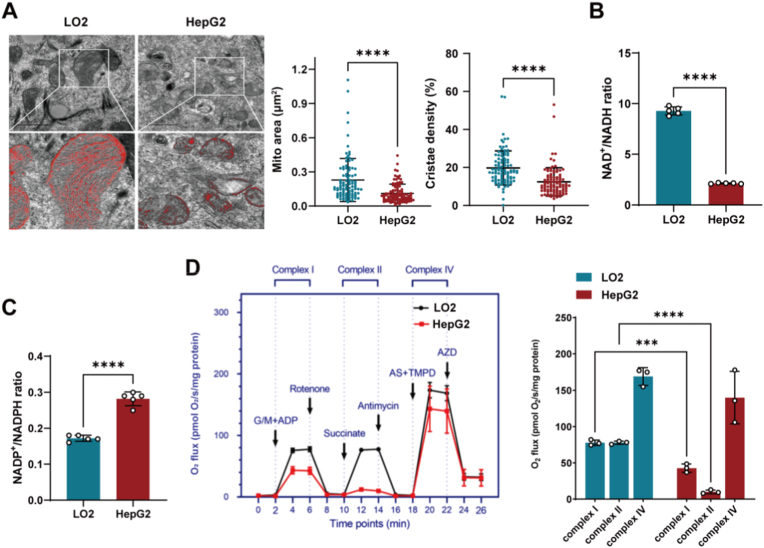
Mitochondrial structural and functional defects in hepatocellular carcinoma cells. **A** TEM analysis of mitochondrial architecture in LO2 versus HepG2 cell lines. Left panels show representative TEM images with magnified insets highlighting cristae structure (red outlines indicate mitochondria). Scale bars: 1 μm. Right panels show quantification of mitochondrial area (μm^2^) and cristae density (%) from n = 100 mitochondria per cell line, pooled from three independent experiments (≥30 mitochondria per experiment). **B,C** NAD^+^/NADH (**B**) and NADP^+^/NADPH (**C**) ratio comparison between isolated mitochondria from LO2 and HepG2 cells lines (n = 5). **D** Respiratory complex activities in isolated mitochondria from LO2 and HepG2 cells, measured by high-resolution respirometry (n = 3). Isolated mitochondria were used to enable direct addition of membrane-impermeable substrates/inhibitors for individual complex assessment. G/M + ADP: glutamate/malate + ADP for Complex I; Rotenone: Complex I inhibitor; Succinate: Complex II substrate; Antimycin: Complex III inhibitor; AS + TMPD: ascorbate + TMPD for Complex IV; AZD: azide (Complex IV inhibitor). Right panel: Quantified oxygen consumption rates for Complex I- and II-dependent respiration, and Complex IV activity. All statistical data represent mean ± SD, analyzed using unpaired Student's t-test (A-C), and two-way ANOVA (D). ∗∗∗P < 0.001, ∗∗∗∗P < 0.0001.

For isolated mitochondria experiments (Fig. 1D), mitochondria were isolated from LO2 and HepG2 cells, and equal protein loading was confirmed by VDAC Western blot. Mitochondria were resuspended in MiR05 respiration medium at 37 °C, and 2 ml of mitochondrial suspension was added to each O2k chamber. Sequential additions were performed as follows: glutamate (10 mM) plus malate (2 mM) with ADP (2.5 mM) to measure Complex I-dependent respiration; rotenone (0.5 μM) to inhibit Complex I; succinate (10 mM) to assess Complex II-dependent respiration; antimycin A (2.5 μM) to block Complex III; and ascorbate (2 mM) plus TMPD (0.5 mM) to determine Complex IV activity, followed by azide (AZD, 10 mM) for Complex IV inhibition. Substrate and inhibitor concentrations were based on established SUIT protocols [30].

For intact cell experiments (Fig. 7D), HepG2 cells stably expressing PHB2-WT, PHB2–Y34E/Y77E, or PHB2–Y34F/Y77F were harvested and resuspended at 1 × 10^6^ cells in 2 mL serum-free MiR05 buffer to avoid potential binding of hydrophobic compounds to serum proteins. Equal mitochondrial content among the three groups was confirmed by VDAC Western blot. All inhibitors and uncouplers were titrated prior to final measurements. Sequential additions were: oligomycin (2.5 μM, ATP synthase inhibitor), FCCP (1 μM, mitochondrial uncoupler), rotenone (0.5 μM, Complex I inhibitor), and antimycin A (2.5 μM, Complex III inhibitor). Oxygen consumption rates were calculated using DatLab software and normalized to protein content.

### 2.9 L-lactate production assay

Extracellular l-lactate levels were measured using the l-Lactate Assay Kit (Beyotime Biotechnology) according to the manufacturer's protocol. Briefly, culture supernatants (50 μl) from HepG2 cells expressing PHB2-WT, PHB2–Y34E/Y77E, or PHB2–Y34F/Y77F were collected and mixed with 50 μl working solution (containing Enzyme Solution, Substrate, and Lactate Assay Buffer). After incubation at 37 °C for 30 min, absorbance was measured at 450 nm. l-lactate concentrations were calculated from a standard curve (0-0.5 mM) and normalized to cell number.

### 2.10 NAD^+^/NADH ratio measurement

NAD^+^/NADH ratios were determined using a well-established enzymatic spectrophotometric method based on NADH absorption at 340 nm [31]. Approximately 1 × 10^6^ cells were harvested and divided into two portions for whole-cell analysis, or mitochondria were isolated from an equivalent number of cells. For NAD^+^ extraction, cells or isolated mitochondria were resuspended in 200 μl of 0.2 M NaOH, while for NADH extraction, cells or isolated mitochondria were resuspended in 200 μl of 0.2 M HCl. Both samples were heated at 100 °C for 10 min, cooled on ice, and neutralized with equal volumes of 0.2 M HCl or 0.2 M NaOH, respectively. After centrifugation at 12,000 g for 10 min at 4 °C, supernatants were collected for analysis.

For NAD^+^ quantification, 50 μl of alkaline extract was mixed with 500 μl reaction buffer (100 mM glycine-NaOH pH 8.8, 0.5% BSA) and 50 μl of 10% ethanol. Baseline absorbance at 340 nm was recorded, then 10 μl of yeast alcohol dehydrogenase (10 U/ml) was added. The reaction proceeded at 25 °C until absorbance reached a plateau (10-15 min). For NADH quantification, 50 μl of acid extract was mixed with 500 μl reaction buffer and absorbance at 340 nm was measured directly. Standard curves were generated using NAD^+^ and NADH standards ranging from 0 to 200 μM, with correlation coefficients exceeding 0.99. Sample concentrations were calculated from standard curves, corrected for dilution factors, and normalized to cell number. The NAD^+^/NADH ratio was calculated by dividing NAD^+^ concentration by NADH concentration.

### 2.11 NADP^+^/NADPH ratio measurement

Cellular NADP^+^ and NADPH levels were determined using an enzymatic cycling assay based on glucose-6-phosphate dehydrogenase (G6PDH) activity [32]. Briefly, approximately 1 × 10^6^ cells or isolated mitochondria were homogenized in 200 μl ice-cold extraction buffer (0.1 M Tris-HCl pH 8.0, 0.01 M EDTA, 0.05% Triton X-100). The homogenate was divided into two aliquots for separate measurement of total NADP(H) (NADP^+^ + NADPH) and NADPH alone.

For NADPH measurement, one aliquot (100 μl) was heated at 60 °C for 30 min to selectively degrade NADP^+^, then cooled on ice and centrifuged at 12,000 g for 5 min at 4 °C. For total NADP(H), the other aliquot was processed without heating. Supernatants (20 μl) from each were added to 96-well plates containing 180 μL reaction mixture (0.1 M Tris-HCl pH 8.0, 5 mM glucose-6-phosphate, 1 mM MgCl_2_, 0.5 U/mL G6PDH from baker's yeast (Sigma-Aldrich), and 0.2 mM MTT as chromogenic substrate). The reaction was incubated at 37 °C for 30 min, and absorbance was measured at 570 nm using a microplate reader (Bio-Rad).

NADPH concentrations were calculated from a standard curve (0-100 μM NADPH). NADP^+^ levels were determined by subtracting NADPH from total NADP(H). The NADP^+^/NADPH ratio was calculated accordingly.

### 2.12 krCRISPR for generating conditional knockout cells

Our study introduces krCRISPR, an efficient strategy for generating conditional knockout cells using double episomal vectors [33]. First, we modified the epiCRISPR plasmid to express SpCas9 and tTA nuclease from the EF1α promoter using self-cleaving P2A peptides, while expressing gRNA from the human U6 promoter. Synthetic oligonucleotide duplexes encoding gRNAs can be easily cloned into *Bsp*QI restriction sites. We then further constructed a rescue plasmid containing copGFP and puromycin resistance genes co-expressed from a pTRE promoter and exogenous PHB2 gene downstream of the promoter. Synonymous mutations were introduced into the gRNA targeting sequence to prevent Cas9 cleavage. By co-transfecting both plasmids into cells, the endogenous PHB2 gene was efficiently knocked out, while exogenous expressions of wild-type and mutant PHB2 were induced. To verify the efficiency of the method, we measured cell proliferation, colony formation, and protein expression.

### 2.13 Liposome co-sedimentation assay

Large unilamellar vesicles (LUVs) were composed of 1-palmitoyl-2-oleoyl-sn-glycero-3-phosphocholine (POPC, Avanti #850457), 1-palmitoyl-2-oleoyl-sn-glycero-3-phosphoethanolamine (POPE, Avanti #850757), and 1,1′,3,3′-bis[1,2-dioleoyl-sn-glycero-3-phospho]-sn-glycerol (TOCL, 18:1 cardiolipin, Avanti #710335) at indicated ratios in chloroform. Lipids were mixed and dried under nitrogen, and further desiccated under vacuum for ≥2 h. The lipid film was hydrated in binding buffer (20 mM HEPES-KOH pH 7.4, 150 mM KCl, 1 mM MgCl_2_) at 2 mM total lipid concentration. After 5 freeze-thaw cycles, LUVs were prepared by 21 extrusions through a 100 nm polycarbonate membrane using a Mini-Extruder (Avanti Polar Lipids) at room temperature. The resulting vesicles had a mean diameter of ∼105 nm as confirmed by dynamic light scattering. For concentration-dependent binding assays, liposomes were prepared with varying CL:PC:PE ratios (15:42.5:42.5, 30:35:35, 60:20:20, 90:5:5). Purified GST-tagged PHB1 or PHB2 proteins (wild-type or mutants including R54T, R71T, R54T/R71T, ΔSPFH1 for PHB2; R41T, K63T, R41T/K63T, ΔSPFH1 for PHB1) at 2 μg were incubated with 200 nmol LUVs for 30 min at 25 °C. After ultracentrifugation at 100,000×*g* for 30 min at 4 °C, pellets (P) and supernatants (S) were analyzed by SDS-PAGE and Coomassie staining.

### 2.14 Coarse-grained models of PHB1/2 integrated with lipids

To investigate the dynamic changes of the protein-lipid complex formed by PHB1/2 complex and the mitochondrial inner membrane bilayer, a coarse-grained system described by the Martini force field (v2.2) was generated using CHARMM-GUI. The upper layer of the lipid bilayer consisted of 900 POPC, 700 POPE, and 400 cardiolipin molecules, while the lower layer was composed of 918 POPC, 720 POPE, and 420 cardiolipin molecules. The cardiolipin molecules were modeled using a coarse-grained model containing two negative charges. The simulation temperature was set to 310 K, and the Nosé-Hoover thermostat was used for temperature control. The pressure was set to 1 bar, and the Parrinello-Rahman barostat was used for pressure control. In addition, semi-isotropic coupling was used to control the pressure in the XY and Z directions separately. The Lenard-Jones (LJ) truncation value was set to 1.2 nm. Electrostatic interactions were calculated using the particle mesh Ewald (PME) method. The time step was set to 20 fs, and constraints were applied to the protein backbone to prevent dissociation of oligomers. The simulation time was 4 μs, and the conformation was output every 1 ns. The simulation software was GROMACS 2019.6, and the analysis software included GROMACS_tools, Mdtraj, MDAnalysis, and VMD for visualization.

### 2.15 Analysis of the distribution and density of lipids surrounding polar and positively charged residues in IMM

Radial distribution functions measure the variation of the density of beads as a function of distance from a reference bead relative to their average density in the system. They show whether lipid beads are more or less likely to be found around certain protein residues. They were calculated with the mdtraj python package for lipids (phosphoric acid bead) with respect to their distance from protein residues (main chain bead for glycine, side chain bead for others) over simulations. The radial distribution function (RDF) or pair correlation function gAB (r) between particles of type A (protein residues) and B (phosphoric acid bead) is defined in the following way:

with <ρB(r)> the particle density of type B at a distance r around particles A, and the particle density of type B averaged over all spheres around A particles with radius rmax (1.5 nm).

The density of the lipids (phosphoric acid bead) was calculated by MDAnalysis python package with 5 Å bin size for the density grid. The positions of phospholipids were mapped onto the xy plane, where darker color indicates higher density.

$$\begin{array}{rr} g_{AB}\left(r\right) & =\frac{\left\langle \rho_{B}\left(r\right)\right\rangle }{\langle \rho_{B}\rangle_{local}}\\ & =\frac{1}{\langle \rho_{B}\rangle_{local}}\frac{1}{N_{A}}\sum_{i\in A}\sum_{j\in B}\frac{\delta \left(r_{ij}-r\right)}{4\pi r^{2}} \end{array}$$

*Analysis of PHB1/2-cardiolipin interactions from molecular dynamics simulations*: Temporal interaction dynamics between PHB1/2 complex subunits and cardiolipin were analyzed from the coarse-grained molecular dynamics trajectories. Contact between cardiolipin and PHB residues was defined when cardiolipin headgroup beads were within 0.6 nm of protein residue beads. For each PHB1 and PHB2 subunit, interactions with cardiolipin were monitored at 1 ns intervals throughout the 4 μs simulation. Key residues including positively charged amino acids (PHB1: K4, R41, K63; PHB2: K6, R17, R54, R71) and polar residues (PHB1: H55; PHB2: Y34, G35, S39, Y77) were analyzed for their interaction frequency with cardiolipin over time. Interaction frequency was calculated as the fraction of simulation time that each residue maintained contact with cardiolipin, and visualized using color gradients representing low (blue) to high (red) interaction frequencies. The analysis was performed separately for inner and outer leaflet interactions to distinguish membrane-facing versus matrix-facing cardiolipin binding.

### 2.16 Cell proliferation assay

Cell proliferation was assessed using the Click-iT EdU Alexa Fluor 488 Imaging Kit (Invitrogen). Cells were incubated with 10 μM EdU for 2 h, fixed with 4% paraformaldehyde, permeabilized, and subjected to click chemistry reaction according to the manufacturer's protocol. Nuclei were counterstained with DAPI. Images were acquired using a Zeiss LSM 880 confocal microscope, and EdU-positive cells were quantified from at least 500 cells per condition.

### 2.17 Cell viability assay

Cell viability was assessed by flow cytometry using 4′,6-diamidino-2-phenylindole (DAPI) staining to detect dead or dying cells with compromised membrane integrity. Briefly, HepG2 cells stably expressing PHB2-WT, PHB2–Y34E/Y77E, or PHB2–Y34F/Y77F were harvested by trypsinization and resuspended in phosphate-buffered saline (PBS) at a density of 1 × 10^6^ cells/mL. Cells were incubated with 1 μg/mL DAPI (Sigma-Aldrich, D9542) for 5 min at room temperature in the dark. Unstained cells served as negative controls for gating.

Flow cytometry analysis was performed using a CytoFlex flow cytometer (Beckman Coulter). A minimum of 10,000 events per sample were acquired. Data were analyzed using CytExpert software (version 2.4, Beckman Coulter). DAPI-positive cells were gated based on fluorescence intensity in the Violet-450 channel (excitation 405 nm, emission 450 nm), with viability calculated as the percentage of DAPI-negative cells.

### 2.18 Cardiolipin content measurement

Cardiolipin content was quantified using the Cardiolipin Green Fluorescent Staining Kit (NAO) (Beyotime Biotechnology, Cat# C1872S) according to the manufacturer's instructions with minor modifications for mitochondrial extracts. The NAO assay, while widely used for cardiolipin quantification, has known limitations such as low solubility, weak fluorescence, and self-quenching, which can lead to a 15-25% decrease in signal intensity within 20 min. Measurements were performed immediately after staining to minimize these effects.

Briefly, mitochondria were isolated from LO2 and HepG2 cells (or HepG2 cells stably expressing PHB2-WT, PHB2–Y34E/Y77E, or PHB2–Y34F/Y77F) by differential centrifugation. Cells were harvested at 70-80% confluence, washed twice with ice-cold PBS, and resuspended in mitochondrial isolation buffer (250 mM sucrose, 20 mM HEPES pH 7.4, 10 mM KCl, 1.5 mM MgCl_2_, 1 mM EDTA, 1 mM EGTA, supplemented with protease inhibitors). Cells were homogenized using a Dounce homogenizer (20-30 strokes on ice), and nuclei/unbroken cells were pelleted at 800×*g* for 10 min at 4 °C. The supernatant was centrifuged at 12,000×*g* for 15 min at 4 °C to obtain the mitochondrial pellet, which was washed once in isolation buffer and resuspended in assay buffer.

Mitochondrial content was normalized across samples by adjusting input cell numbers based on total protein concentration (determined by BCA assay) and confirmed by Western blot analysis of VDAC (voltage-dependent anion channel) as a mitochondrial loading control. Equivalent mitochondrial aliquots (typically 50-100 μg protein per sample) were diluted to 100 μL in assay buffer and incubated with 100 nM NAO working solution (prepared fresh from the kit stock) for 20 min at 37 °C in the dark. Fluorescence intensity was measured using a microplate reader (excitation: 495 nm, emission: 519 nm). Background fluorescence from unstained controls was subtracted, and data were expressed as relative fluorescence units (RFU) normalized to mitochondrial protein content (RFU/mg protein).

For in vivo xenograft tumors, tissues were harvested at day 25 post-injection. Tumors and ipsilateral liver (paired normal controls) were homogenized in mitochondrial isolation buffer. Mitochondrial content was normalized across samples by adjusting homogenate loading, confirmed by VDAC Western blot. Cardiolipin content was quantified using the NAO assay as above, with fluorescence normalized to the PHB2-WT normal liver group (set as 100%).

### 2.19 Xenograft tumor model

All animal procedures were approved by the Animal Ethics Review Committee of Zhejiang University and performed in accordance with the guidelines of NIH Publication No. 85-23 (revised 1996) (approved number: AIRB-2023-1448). Six-week-old male BALB/c nude mice were subcutaneously injected with 5 × 10^6^ HepG2 cells stably expressing PHB2-WT, PHB2–Y34E/Y77E, or PHB2–Y34F/Y77F in 100 μl PBS mixed with Matrigel (1:1). Tumor volumes were measured every 3 days using calipers and calculated as (length × width^2^)/2. Mice were sacrificed at day 25, and tumors were excised, weighed, and photographed.

### 2.20 Statistical analysis

Data are presented as mean ± standard deviation (SD) from at least three independent biological replicates. Statistical analyses were performed using GraphPad Prism 9.0 (GraphPad Software). Normality of data distribution was assessed using the Shapiro-Wilk test, and homogeneity of variance was evaluated using Levene's test. For normally distributed data with equal variances, comparisons between two groups were analyzed using unpaired two-tailed Student's t-test, and multiple group comparisons were performed using one-way or two-way analysis of variance (ANOVA) followed by Bonferroni's post-hoc test for pairwise comparisons. For data that did not meet parametric assumptions, Mann-Whitney *U* test (two groups) or Kruskal-Wallis test with Dunn's post-hoc correction (multiple groups) was applied. Kaplan-Meier survival analysis was performed using TCGA-LIHC (Liver Hepatocellular Carcinoma) dataset with log-rank (Mantel-Cox) test for comparison between high and low expression groups, stratified by median expression. P < 0.05 was considered statistically significant.

## 3 Results

### 3.1 Hepatocellular carcinoma cells exhibit severe mitochondrial cristae disorganization and OXPHOS dysfunction

To investigate mitochondrial structural and functional alterations in hepatocellular carcinoma, we performed comprehensive ultrastructural and metabolic analyses comparing normal hepatocyte cell line LO2 with hepatocellular carcinoma cell line HepG2. Transmission electron microscopy revealed profound mitochondrial cristae abnormalities in HepG2 cells (Fig. 1A). While LO2 cells displayed well-organized cristae with regular lamellar structures, HepG2 cells exhibited markedly disrupted cristae architecture with reduced density and irregular, fragmented cristae membranes. Additionally, HepG2 mitochondria showed increased cross-sectional area, consistent with mitochondrial swelling often observed in metabolically compromised cells.

These structural abnormalities were accompanied by significant metabolic dysfunction. The NAD^+^/NADH ratio was reduced in both whole cells and isolated mitochondria of HepG2 cells compared to LO2 cells(Fig. 1B, and Supplemental Fig. 1A). This suggested impaired oxidative phosphorylation and a shift toward reductive metabolism, characteristic of cancer cell redox imbalance [34]. Furthermore, we measured NADP^+^/NADPH ratios in whole cells and isolated mitochondria. HepG2 cells showed significantly higher ratios than LO2 cells in both fractions (Fig. 1C, and Supplemental Fig. 1B). This suggested increased NADPH consumption, potentially supporting antioxidant defense and biosynthetic demands in HCC cells [35].

Consistent with these redox alterations, high-resolution respirometry of isolated mitochondria from LO2 and HepG2 cells revealed severe defects in Complex I- and Complex II-dependent respiration and Complex IV activity, with reduced function in HepG2 (Fig. 1D). Furthermore, cardiolipin content, which is essential for cristae organization and respiratory complex stability, was approximately 45% lower in HepG2 cells than in LO2 cells (Supplementary Fig. S1C and D) [36,37]. Together, these findings establish that hepatocellular carcinoma cells exhibit coordinated mitochondrial structural and functional defects, linking cristae disorganization to oxidative phosphorylation impairment and altered redox homeostasis.

### 3.2 PHB2 undergoes specific Src-mediated tyrosine phosphorylation in hepatocellular carcinoma cells

**Fig. 2:**
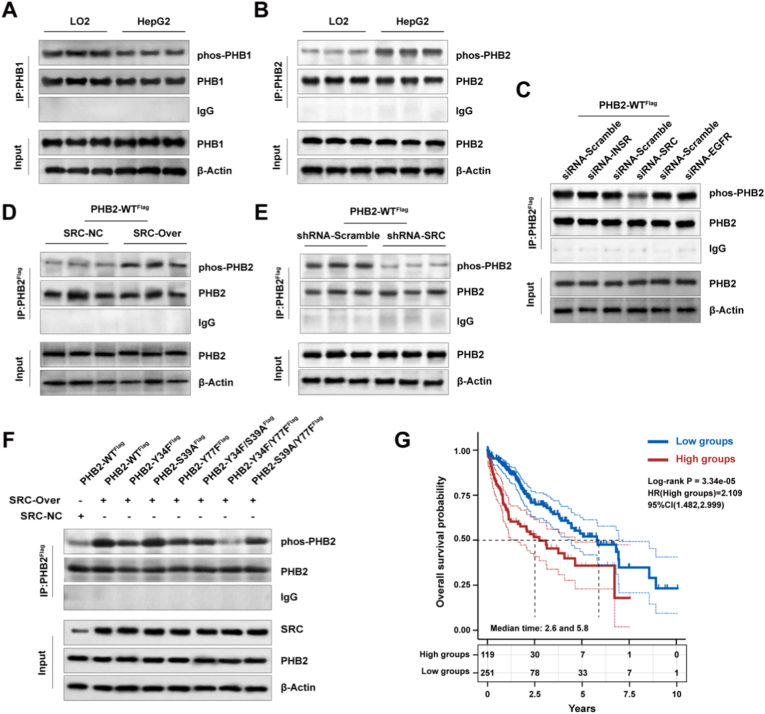
Src kinase specifically phosphorylates PHB2 at Y34 and Y77 in hepatocellular carcinoma cells. **A,B** Differential phosphorylation of PHB proteins in LO2 versus HepG2 cell lines. (**A**) PHB1 phosphorylation analysis. (**B**) PHB2 phosphorylation analysis. Upper panels: Immunoprecipitation (IP) with *anti*-PHB1 or *anti*-PHB2 antibodies followed by detection of phosphorylated proteins (phos-PHB1/2) and total PHB1/2. IgG serves as negative control. Lower panels: Input lysates showing total PHB1/2 and β-actin loading control. **C** Identification of kinases involved in PHB2 phosphorylation in HepG2 cells. HepG2 cells expressing PHB2-WTFlag were transfected with siRNA targeting INSR, SRC, or EGFR, or scramble control. Upper panel: IP with *anti*-PHB2 antibody followed by Western blot detection of phos-PHB2, total PHB2, and IgG control. Lower panel: Input lysates showing PHB2 and β-Actin (loading control). **D** Effect of SRC overexpression on PHB2 phosphorylation in HepG2 cells. HepG2 cells expressing PHB2-WT^Flag^ were transduced with control (SRC-NC) or SRC-overexpressing lentivirus. IP-Western blot analysis as described in (B). **E** Effect of SRC knockdown on PHB2 phosphorylation in HepG2 cells. HepG2 cells expressing PHB2-WT^Flag^ were transfected with scramble control or SRC-specific shRNA. IP-Western blot analysis as described in (B). **F** Identification of SRC phosphorylation sites on PHB2 in HepG2 cells. HepG2 cells were transfected with various Flag-tagged PHB2 mutants (Y34F, S39A, Y77F, Y34F/S39A, Y34F/Y77F, S39A/Y77F) with or without SRC overexpression. IP-Western blot analysis shows phosphorylation levels of different PHB2 mutants. All immunoprecipitation and Western blot experiments were performed in at least three independent experiments with similar results. Representative blots are shown. **G** The KM survival curve of the Src gene in TCGA data, where different groups are tested using the log-rank test. HR (High exp) represents the hazard ratio of the high expression group relative to the low expression group.

The maintenance of mitochondrial cristae architecture critically depends on the prohibitin 1/2 (PHB1/2) complex, which forms ring-like structures anchored to the inner membrane through lipid interactions [16]. Given that PHB1/2 dysfunction leads to cristae collapse similar to our observations in HepG2 cells, we investigated whether PHB1/2 proteins undergo aberrant post-translational modifications in hepatocellular carcinoma. Immunoprecipitation followed by phosphotyrosine immunoblotting revealed that PHB2, but not PHB1, exhibited significantly elevated tyrosine phosphorylation in HepG2 cells compared to LO2 cells. Importantly, total PHB1 and PHB2 protein levels remained unchanged between the two cell lines, indicating that the increased PHB2 phosphorylation represents a specific regulatory event rather than a consequence of altered protein abundance (Fig. 2A and B).

To identify the kinase responsible for PHB2 phosphorylation, we performed targeted siRNA screening of tyrosine kinases known to be dysregulated in hepatocellular carcinoma. Among the candidates tested (insulin receptor INSR, Src kinase, and epidermal growth factor receptor EGFR), only Src knockdown significantly reduced PHB2 phosphorylation in HepG2 cells (Fig. 2C). The essential role of Src in mediating PHB2 phosphorylation was further validated through gain- and loss-of function experiments. Src overexpression markedly enhanced PHB2 phosphorylation (Fig. 2D), while stable Src knockdown using shRNA substantially decreased phosphorylation levels (Fig. 2E), confirming that Src is both necessary and sufficient for PHB2 tyrosine phosphorylation in hepatocellular carcinoma cells.

To precisely map the Src phosphorylation sites on PHB2, we 392 generated a panel of tyrosine-to-phenylalanine and serine-to-alanine point mutants targeting predicted phosphorylation sites. Systematic mutational analysis revealed that simultaneous mutation of Y34 and Y77 to phenylalanine (Y34F/Y77F) completely abolished Src-mediated PHB2 phosphorylation, while individual mutations or combinations targeting other residues retained phosphorylation capability (Fig. 2F). These results identify Y34 and Y77 as the primary Src phosphorylation sites on PHB2.

Bioinformatics analysis provided further support for Y34 and Y77 as Src phosphorylation sites. Scansite 4.0 kinase recognition motif analysis revealed that both Y34 and Y77 exhibit high confidence scores (>0.75) for Src kinase recognition, with Y34 showing particularly strong consensus with the Src recognition motif (Supplemental Fig. 2A). Integrated prediction using five independent algorithms (NetPhos 3.1, GPS 5.0, KinasePhos 2.0, PhosphoBlast, and MusiteDeep) consistently identified Y34 and Y77 as high-probability Src phosphorylation sites, with consensus scores exceeding 0.8 across all methods (Supplemental Fig. 2B). Evolutionary conservation analysis using ConSurf demonstrated that Y34 and Y77 are highly conserved across vertebrate species, suggesting functional importance of these residues (Supplemental Fig. 2C). Additionally, PHB2 N-terminal positively charged and polar amino acids critical for cardiolipin binding showed strong evolutionary conservation (Supplemental Fig. 2D), consistent with their essential role in membrane anchoring. Functional impact prediction algorithms (SIFT and PolyPhen-2) indicated that Y34F and Y77F mutations would significantly affect PHB2 protein function, supporting the functional importance of these tyrosine residues (Supplemental Fig. 2E).

The clinical relevance of this finding is underscored by The Cancer Genome Atlas (TCGA, https://tcga-data.nci.nih.gov/docs/publications/tcga/) database analysis showing that high SRC expression correlates with significantly worse overall survival in hepatocellular carcinoma patients (Fig. 2G), suggesting that 419 the Src-PHB2 axis may contribute to disease progression.

### 3.3 Src-mediated phosphorylation disrupts PHB1/2 complex stability and triggers PHB2 cytoplasmic mislocalization

**Fig. 3:**
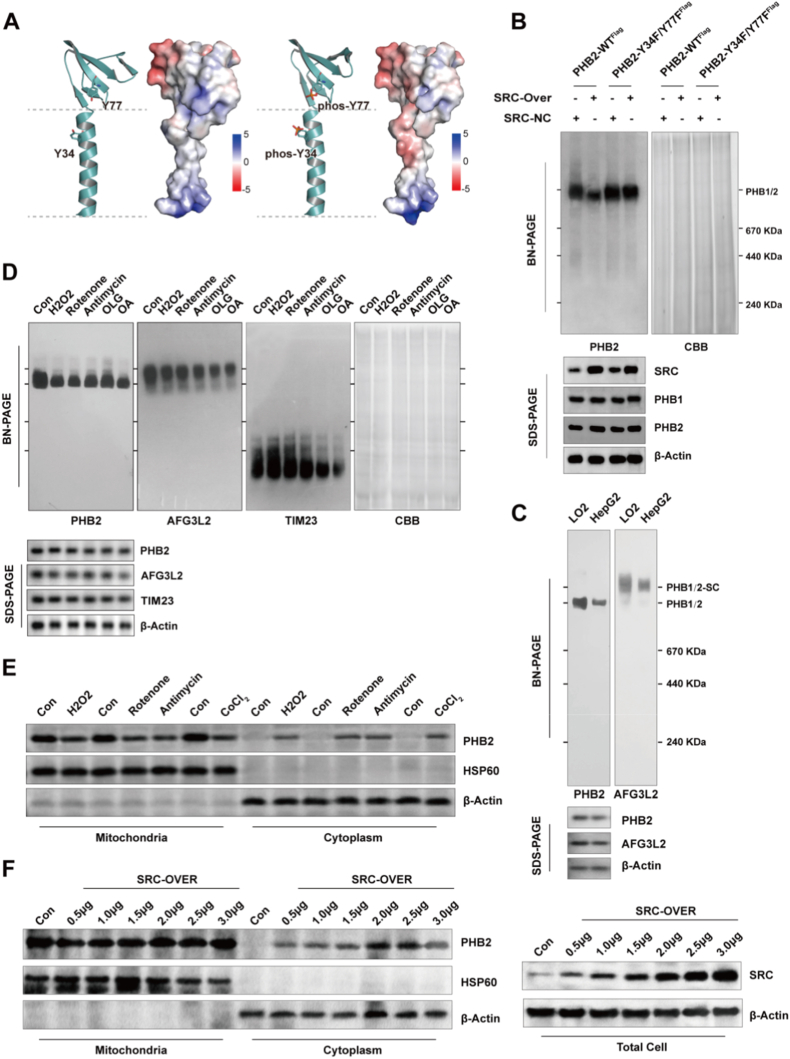
Src-mediated phosphorylation disrupts PHB1/2 complex stability and triggers PHB2 cytoplasmic mislocalization. **A** Electrostatic surface potential analysis of PHB2 showing the effect of phosphorylation. Left panel: wild-type PHB2 with Y34 and Y77 residues. Right panel: phosphorylated PHB2 (phos-Y34 and phos-Y77) showing altered electrostatic distribution. Blue indicates positive charge (+5), white is neutral (0), and red indicates negative charge (−5). The double-framed line represents the inner mitochondria membrane. **B** Effect of Src-mediated phosphorylation on PHB1/2 complex formation. Blue Native PAGE (BN-PAGE) analysis of PHB2-WT and phosphorylation-resistant mutant PHB2–Y34F/Y77F^Flag^ in HepG2 cells with or without SRC overexpression. Protein load was verified by Coomassie blue (CBB) staining. Lower panel shows SDS-PAGE verification of SRC, PHB1, PHB2, and β-Actin expression. **C** Analysis of PHB1/2 complex formation in LO2 versus HepG2 cell lines. Upper panel: representative BN-PAGE showing PHB1/2-SC (supercomplex) and PHB1/2 complex formation detected with PHB2 and AFG3L2 antibodies. Lower panel: SDS-PAGE Western blot analysis of PHB2, AFG3L2, and β-Actin (loading control) expression levels. **D** Effect of OXPHOS inhibitors on PHB1/2 complex formation. Left panels: representative BN-PAGE analysis of PHB1/2-SC and PHB1/2 complexes in control and treated conditions (H_2_O_2_, Rotenone, Antimycin, Oligomycin (OLG), and combined Antimycin/Oligomycin (OA)). Protein load was verified by Coomassie blue (CBB) staining. Lower panel: representative Western blot showing protein expression levels of PHB2, AFG3L2, TIM23, and β-Actin (loading control). **E** Subcellular distribution of PHB2 under tumor microenvironment stresses. Western blot analysis of PHB2 in mitochondrial and cytoplasmic fractions of HepG2 cells treated with H_2_O_2_ (oxidative stress), CoCl_2_ (hypoxia mimetic), rotenone (Complex I inhibitor), or antimycin (Complex III inhibitor). **F** Dose-dependent PHB2 redistribution in response to increasing SRC expression levels. Western blot analysis showing PHB2 subcellular localization in mitochondrial and cytoplasmic fractions after SRC overexpression in HepG2 cells. HSP60 serves as mitochondrial marker and β-Actin as cytoplasmic marker (E, F). All Blue Native PAGE and Western blot experiments (B–F) were performed in at least three independent experiments. Representative images are shown.

Having established that Src phosphorylates PHB2 at Y34 and Y77, we investigated the functional consequences of this modification on PHB complex integrity and subcellular localization. Structural modeling revealed that Y34 and Y77 residues are positioned near the membrane-binding interface of PHB2. Electrostatic surface potential analysis demonstrated that phosphorylation of these tyrosines introduces negative charges in a region normally characterized by positive electrostatic potential, creating unfavorable electrostatic repulsion with the negatively charged inner mitochondrial membrane phospholipids (Fig. 3A).

Blue Native PAGE analysis revealed that Src overexpression dramatically reduced both PHB1/2 supercomplex and PHB1/2 complex levels in cells expressing wild-type PHB2, but had no effect in cells expressing the phosphorylation-resistant PHB2–Y34F/Y77F mutant (Fig. 3B). This demonstrates that Y34/Y77 phosphorylation is both necessary and sufficient for Src-induced complex disassembly. Consistent with these findings, HepG2 cells displayed markedly reduced PHB1/2 complex levels compared to LO2 cells, with correspondingly decreased incorporation of the PHB-interacting *m*-AAA protease AFG3L2 into the complex (Fig. 3C).

The PHB1/2 complexes showed remarkable sensitivity to mitochondrial stress conditions that simulate the tumor microenvironment. Treatment with oxidative stress (H2O2), Complex I inhibition (rotenone), Complex III inhibition (antimycin), or ATP synthase inhibition (oligomycin) all resulted in PHB1/2 complex dissociation, while the unrelated TIM23 translocase complex remained stable under identical conditions (Fig. 3D). This selective vulnerability suggests that PHB1/2 complexes serve as sensors of mitochondrial dysfunction.

Subcellular fractionation assays revealed that multiple stress stimuli triggered increased cytoplasmic accumulation of prohibitin 2 (PHB2) in HepG2 cells, accompanied by a corresponding reduction in its mitochondrial pool. Specifically, treatment with H_2_O_2_ (oxidative stress inducer), CoCl_2_ (hypoxia mimetic), rotenone, or antimycin all recapitulated this PHB2 cytoplasmic translocation, which mimicks the stressed tumor microenvironment (Fig. 3E). Dose-response analyses further showed that escalating Src expression levels drove progressive PHB2 redistribution, with more pronounced cytoplasmic accumulation observed at higher Src abundance (Fig. 3F). This dose-dependent association between Src expression and PHB2 mislocalization directly links the extent of PHB2 cytoplasmic accumulation to Src kinase activity.

### 3.4 PHB1/2 complexes specifically recognize cardiolipin through conserved positively charged residues

**Fig. 4:**
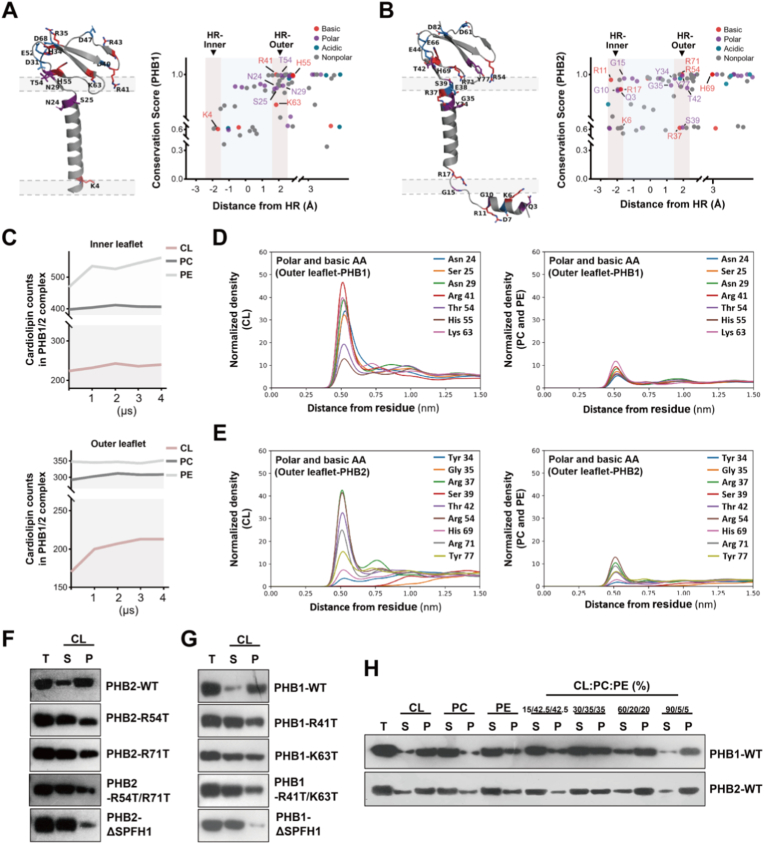
PHB1/2 complexes interact specifically with cardiolipin through conserved positively charged residues. **A,B** Structural analysis of PHB-cardiolipin interaction. Left panel: PHB1 structure showing key positively charged residues (K4, R41, K63) and polar residues involved in cardiolipin binding (**A**). PHB2 structure highlighting key positively charged residues (K6, R17, R54, R71) and polar residues involved in cardiolipin binding (**B**). Right panel: Conservation score analysis showing the distribution of basic, polar, acidic, and nonpolar amino acids relative to their distance from the head region (HR) of cardiolipin on both inner and outer leaflets of the inner mitochondrial membrane (IMM). **C** Coarse-grained molecular dynamics simulation showing temporal changes in phospholipid distribution within the PHB1/2 ring region. Cardiolipin (CL), phosphatidylcholine (PC), and phosphatidylethanolamine (PE) counts on the inner leaflet (upper) and outer leaflet (lower) of the IMM over 4 μs simulation time. **D,E** Radial distribution function analysis of phospholipid interactions with PHB1 (**D**) and PHB2 (**E**). Left panels show interactions between polar and basic amino acids on the outer leaflet of PHB1/2 with CL. Right panels show normalized density of PC and PE. Peak interactions occur at ∼0.5 nm distance. **F,G** Representative blots of cardiolipin binding assays with PHB2 (**F**) and PHB1 (**G**) mutants. Wild-type and mutant proteins (R54T, R71T, R54T/R71T for PHB2; R41T, K63T, R41T/K63T for PHB1) were incubated with cardiolipin vesicles. PHB2-ΔSPFH1 and PHB1-ΔSPFH1 serve as negative controls. **H** Phospholipid vesicle co-precipitation assays. PHB1-WT and PHB2-WT binding to vesicles with varying cardiolipin content (15:42.5:42.5, 30:35:35, 60:20:20, 90:5:5 ratios of CL:PC:PE). T: total protein, S: supernatant (unbound), P: pellet (bound).

To understand the molecular basis of PHB-membrane interactions and how phosphorylation disrupts these interactions, we performed structural analysis and molecular dynamics simulations. Both PHB1 and PHB2 contain clusters of conserved positively charged residues on their membrane-facing surfaces (PHB1: K4, R41, K63; PHB2: K6, R17, R54, R71) that create favorable electrostatic environments for interaction with negatively charged phospholipids (Fig. 4A and B). Conservation analysis revealed enrichment of basic and polar residues at optimal distances (0.5-1.0 nm) from the membrane interface, suggesting evolutionary preservation of lipid-binding capability (Fig. 4C and Supplemental Fig. 2D).

Coarse-grained molecular dynamics simulations over 4 μs demonstrated progressive and selective enrichment of cardiolipin within the PHB1/2 ring region on both leaflets of the inner mitochondrial membrane, while phosphatidylcholine and phosphatidylethanolamine levels remained relatively constant (Fig. 4C and Supplemental Fig. 3). Radial distribution function analysis confirmed that both PHB1 and PHB2 interact preferentially with cardiolipin, showing sharp peaks at approximately 0.5 nm distance indicative of direct electrostatic interactions, whereas interactions with PC and PE were much weaker and less structured (Fig. 4D and E).

Experimental validation using liposome co-sedimentation assays confirmed the computational predictions. Wild-type PHB1 and PHB2 showed robust cardiolipin binding, while mutations targeting the predicted cardiolipin-binding residues (PHB1: R41T, K63T; PHB2: R54T, R71T) progressively impaired binding, with double mutants showing severe defects (Fig. 4F and G). Both PHB1 and PHB2 proteins displayed concentration-dependent binding that increased with cardiolipin content in liposomes, demonstrating specificity for cardiolipin-enriched membrane domains (Fig. 4H). These results establish that PHB1/2 complexes recognize cardiolipin through specific electrostatic interactions mediated by conserved basic residues.

### 3.5 PHB1/2 complexes drive cardiolipin clustering and membrane invagination

**Fig. 5:**
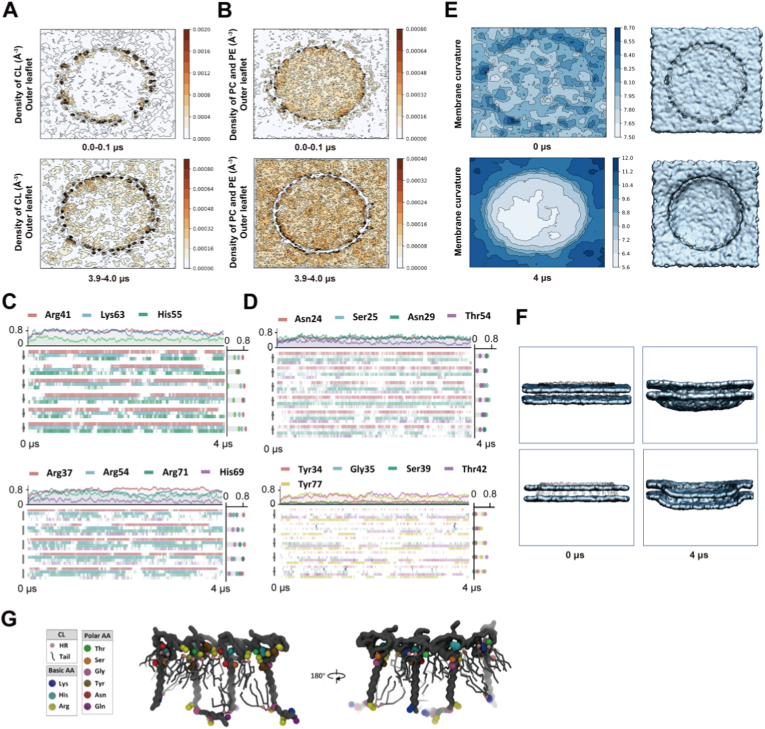
PHB1/2 complexes drive cardiolipin clustering and membrane invagination on the outer leaflet of the inner mitochondrial membrane. **A,B** Density distribution of CL (**A**) and PC/PE (**B**) on the IMM outer leaflet during coarse-grained molecular dynamics simulation. Upper panels show initial distribution at 0-0.1 μs, lower panels show final distribution at 3.9-4.0 μs. Darker colors indicate higher density. The PHB1/2 ring region is indicated by black circles. **C,D** Temporal interaction dynamics between cardiolipin and specific amino acids. (**C**) PHB1 subunits showing interactions with positively charged (Arg41, Lys63) and polar (His55) residues. (**D**) PHB2 subunits showing interactions with positively charged (Asn24, Ser25, Asn29, Thr54) and polar residues (Tyr34, Gly35, Ser39, Thr42, Tyr77). Color gradients indicate interaction frequency over 4 μs simulation. **E** Membrane curvature analysis showing IMM invagination induced by PHB1/2. Blue to white gradient represents membrane height changes from initial flat state (0 μs) to final invaginated state (4 μs), with ∼5.6-8.0 Å depression within the PHB1/2 ring region. **F** Cross-sectional views of membrane curvature evolution. Left panels show initial flat membrane state (0 μs), right panels show invaginated membrane after PHB1/2-induced cardiolipin aggregation (4 μs). **G** Molecular model showing PHB1/2-mediated membrane reorganization. The 180° rotation view illustrates cardiolipin enrichment (colored spheres) within the PHB1/2 ring region after simulation.

Having established the molecular basis of PHB-cardiolipin interactions, we investigated the functional consequences of these interactions on membrane organization. Molecular dynamics simulations revealed dramatic cardiolipin reorganization on the outer leaflet of the inner mitochondrial membrane. While cardiolipin was initially uniformly distributed, it progressively clustered within and around the PHB1/2 ring region, reaching enrichment by 4 μs (Fig. 5A). In contrast, PC and PE maintained relatively uniform distributions throughout the simulation (Fig. 5B), demonstrating the specificity of PHB-mediated lipid reorganization.

Temporal interaction analysis revealed sustained contacts between cardiolipin molecules and specific PHB residues. Notably, PHB2 residues Y34 and Y77 (the Src phosphorylation sites) showed high interaction frequencies with cardiolipin, providing structural context for phosphorylation of these residues disrupting membrane binding (Fig. 5C and D). The PHB-mediated cardiolipin clustering had profound effects on membrane topology. Membrane curvature analysis revealed progressive invagination specifically within the PHB1/2 ring region over the simulation time course (Fig. 5E and F). This localized membrane deformation, occurring selectively where cardiolipin accumulated, suggests that PHB1/2 complexes sculpt membrane architecture through lipid-mediated mechanisms. Three-dimensional visualization confirmed the formation of cardiolipin-enriched microdomains beneath the PHB ring, potentially serving as nucleation sites for cristae formation (Fig. 5G).

### 3.6 PHB2 N-terminal residues regulate cristae structure through the OMA1-OPA1 proteolytic axis

**Fig. 6:**
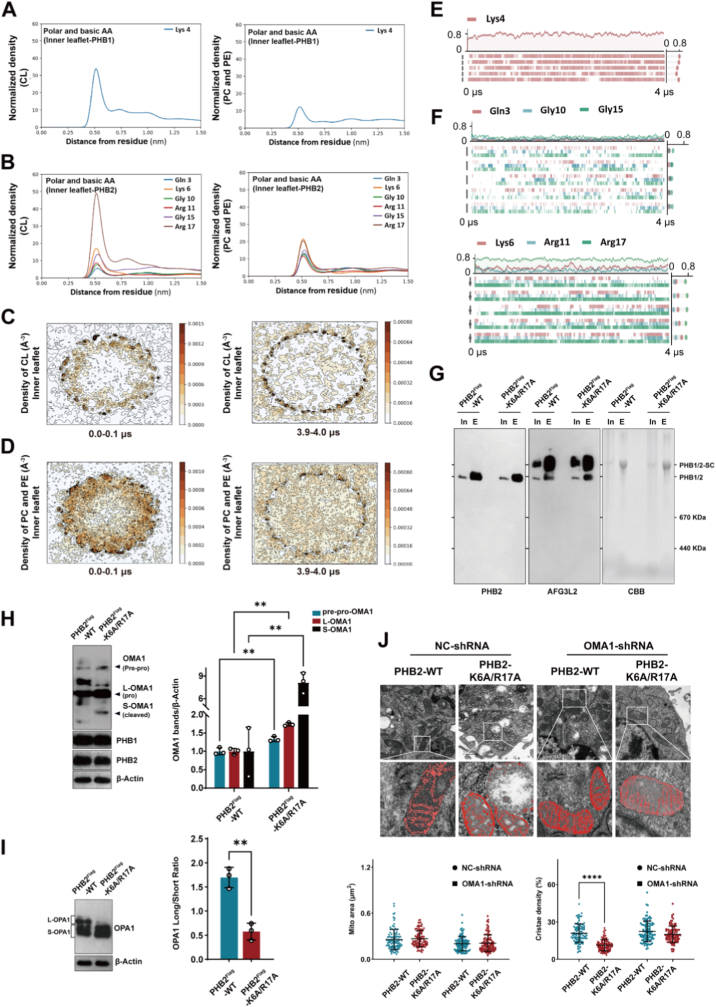
PHB2 N-terminal residues mediate cardiolipin enrichment on the inner leaflet to regulate the OMA1-OPA1 proteolytic axis and cristae structure. **A,B** Radial distribution function analysis of phospholipid interactions on the IMM-inner leaflet. (**A**) PHB1 N-terminal residues (Lys4) and (**B**) PHB2 N-terminal residues (Gln3, Lys6, Gly10, Arg11, Gly15, Arg17) showing preferential interaction with cardiolipin versus PC/PE. Peak interactions occur at ∼0.5 nm distance. **C,D** Temporal evolution of phospholipid density distribution on the IMM-inner leaflet. (**C**) Initial state (0-0.1 μs) and (**D**) final state (3.9-4.0 μs) showing cardiolipin enrichment (upper panels) versus PC/PE distribution (lower panels) around the PHB1/2 ring periphery. **E,F** Interaction dynamics between cardiolipin and N-terminal residues. (**E**) PHB1 subunit interactions with Lys4. (**F**) PHB2 subunit interactions with multiple residues (Gln3, Gly10, Gly15, Lys6, Arg11, Arg17). Color gradients indicate interaction frequency over 4 μs simulation. **G** Effect of PHB2–K6A/R17A mutations on PHB1/2 complex formation. HEK293T-PHB2^−/−^ cells expressing Flag-tagged PHB2-WT or PHB2–K6A/R17A were subjected to anti-Flag immunoprecipitation and elution, followed by Blue Native PAGE analysis to detect PHB1/2 and PHB1/2-SC. In: Input; E: Elution. Protein load was verified by Coomassie blue (CBB) staining. **H,I** OMA1 and OPA1 processing analysis. (**H**) Western blot showing OMA1 isoforms (pre-pro-OMA1, L-OMA1, S-OMA1) in cells expressing PHB2-WT or PHB2–K6A/R17A. Quantification shown in bar graph (n = 3). (**I**) OPA1 cleavage analysis showing L-OPA1 and S-OPA1 ratios with quantification (n = 3). **J** TEM analysis of mitochondrial cristae structure. Representative images and quantification of mitochondrial area and cristae density in HEK293T cells expressing PHB2-WT or PHB2–K6A/R17A, combined with control (NC-shRNA) or OMA1 knockdown (OMA1-shRNA). n = 100 mitochondria per group, pooled from three independent experiments. Scale bars: 1 μm. All statistical data represent mean ± SD, analyzed using unpaired Student's t-test (H, I), and two-way ANOVA (J). ∗∗P < 0.01, ∗∗∗∗P < 0.0001.

While the outer leaflet interactions establish PHB1/2 complex positioning, we discovered that PHB proteins also organize cardiolipin on the inner (matrix-facing) leaflet through their N-terminal residues. Radial distribution function analysis identified strong preferential interactions between cardiolipin and N-terminal basic residues of both PHB1 (particularly K4) and PHB2 (particularly K6 and R17) on the inner leaflet (Fig. 6A and B). Molecular dynamics simulations confirmed progressive cardiolipin enrichment on the inner leaflet periphery of the PHB ring, creating cardiolipin-rich domains (Fig. 6C–F).

**Fig. 7:**
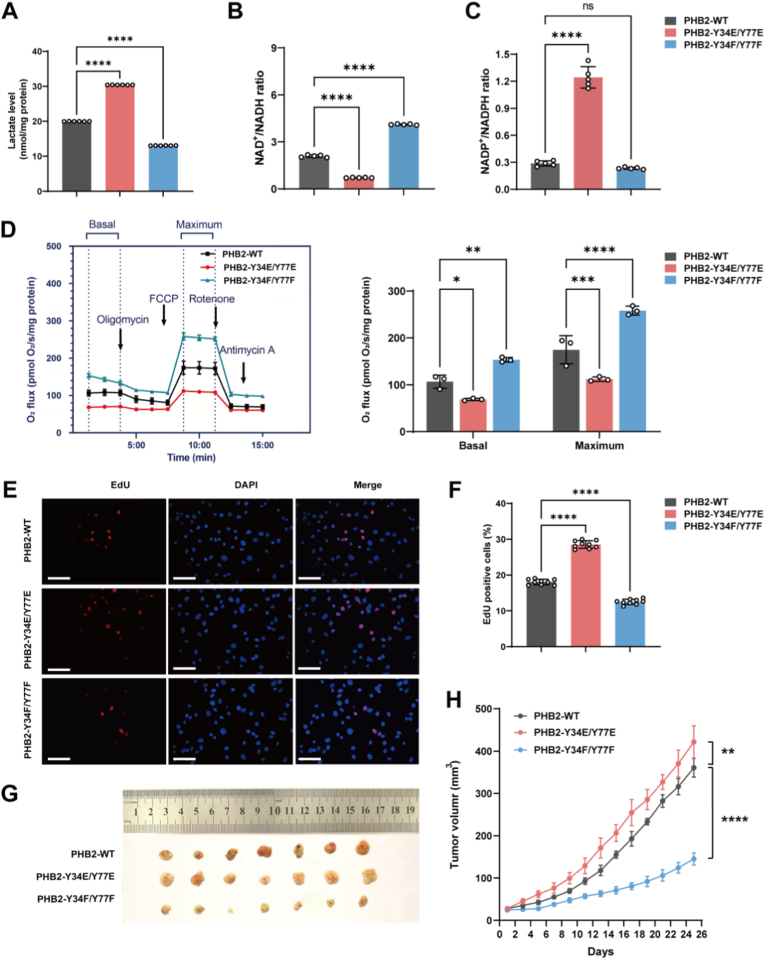
PHB2 phosphorylation at Y34/Y77 sites drives metabolic change and accelerates hepatocellular carcinoma progression. **A** Lactate production analysis in HepG2 cells. Extracellular lactate levels measured in cells expressing PHB2-WT, phosphomimetic PHB2–Y34E/Y77E (mimicking constitutive phosphorylation), or phosphorylation-resistant PHB2–Y34F/Y77F (preventing phosphorylation) (n = 6). **B,C** NAD^+^/NADH (**B**) and NADP^+^/NADPH (**C**) ratio in isolated mitochondria from HepG2 cells expressing PHB2-WT, PHB2–Y34E/Y77E, or PHB2–Y34F/Y77F (n = 5). **D** Cellular respiratory capacity in intact cells. Left panel: Real-time oxygen flux traces showing whole-cell respiration in intact (non-permeabilized) HepG2 cells expressing PHB2-WT, phosphomimetic PHB2–Y34E/Y77E, or phosphorylation-resistant PHB2–Y34F/Y77F. Sequential additions: oligomycin (2.5 μM, ATP synthase inhibitor), FCCP (1 μM, uncoupler for maximal respiration), rotenone (0.5 μM, Complex I inhibitor), and antimycin A (2.5 μM, Complex III inhibitor). Right panel: Quantification of basal and FCCP-stimulated maximal oxygen consumption rates. The phosphomimetic mutant shows impaired respiration while the phosphorylation-resistant mutant shows enhanced respiration (n = 3). Oxygen consumption rates were measured with equal protein loading across all samples, confirmed by VDAC Western blot. **E,F** Cell proliferation analysis using EdU incorporation assay. Representative fluorescence microscopy images showing EdU-positive cells (red) and DAPI nuclear staining (blue) in HepG2 cells expressing PHB2-WT, PHB2–Y34E/Y77E or PHB2–Y34F/Y77F. Scale bars: 50 μm (**E**). Quantification shows increased proliferation with phosphomimetic mutation and decreased proliferation with phosphorylation-resistant mutation (**F**, n = 3). **G** Excised tumors from xenograft mice. Representative images of tumors harvested at day 25. **H** Tumor growth curves. Tumor volume measurements over 25 days in nude mice injected with HepG2 cells expressing PHB2-WT (black), phosphomimetic PHB2–Y34E/Y77E (red), or phosphorylation-resistant PHB2–Y34F/Y77F (blue) (n = 7 mice per group). All statistical data represent mean ± SD, analyzed using one-way ANOVA (A, B, C, F), and two-way ANOVA (D, H). ∗P < 0.05, ∗∗P < 0.01, ∗∗∗P < 0.001, ∗∗∗∗P < 0.0001.

To test the functional importance of these N-terminal interactions, we generated PHB2–K6A/R17A mutants targeting key cardiolipin-binding residues. Notably, this mutation did not impair PHB1/2 complex formation or AFG3L2 incorporation in PHB2-knockout cells reconstituted with the mutant protein (Fig. 6G). Co-immunoprecipitation analysis further showed that the PHB2–K6A/R17A mutation did not disrupt interaction with YME1L1, another *m*-AAA protease involved in mitochondrial quality control (Supplemental Fig. 4A). These findings indicate that the N-terminal basic residues of PHB2 serve a function distinct from complex assembly.

Despite the preserved PHB1/2 complex integrity, the PHB2–K6A/R17A mutation triggered aberrant activation of OMA1, as evidenced by increased S-OMA1 (active form) levels and excessive OPA1 cleavage, resulting in a decreased L-OPA1/S-OPA1 ratio (Fig. 6H and I). These biochemical changes translated into severe cristae disorganization, with reduced cristae density in cells expressing the mutant PHB2. Remarkably, OMA1 knockdown substantially rescued the cristae defects caused by the PHB2–K6A/R17A mutation, restoring cristae density to near-normal levels (Fig. 6J). These results establish that OMA1 hyperactivation is a critical downstream consequence of disrupted PHB2-cardiolipin interactions.

To further test whether OMA1's cardiolipin-binding capacity is required for its regulation by PHB2, we generated an OMA1 mutant lacking the predicted cardiolipin-binding domain (OMA1-Δ148-167). Cells expressing this deletion mutant showed constitutively elevated S-OMA1 levels compared to OMA1-WT, indicating hyperactivation (Supplemental Fig. 4B). Correspondingly, OMA1-Δ148-167 expression resulted in excessive OPA1 cleavage with significantly reduced L-OPA1/S-OPA1 ratio (Supplemental Fig. 4C). TEM analysis confirmed that deletion of the OMA1 cardiolipin-binding domain led to severe cristae disorganization with reduced cristae density (Supplemental Fig. 4D). These results demonstrate that OMA1 hyperactivation is a critical downstream consequence of disrupted PHB2-cardiolipin interactions, linking membrane lipid organization to cristae maintenance through proteolytic regulation of OPA1.

### 3.7 PHB2 phosphorylation promotes glycolytic metabolism and accelerates tumor growth

To establish the functional significance of PHB2 phosphorylation in cancer cell metabolism and tumor progression, we generated HepG2 cell lines stably expressing wild-type PHB2, phosphomimetic PHB2–Y34E/Y77E (mimicking constitutive phosphorylation), or phosphorylation-resistant PHB2–Y34F/Y77F mutants. Flow cytometry of DAPI-positive cells showed similar viability across these PHB2 variants (Supplemental Fig. 5A and B). The phosphomimetic mutant exhibited significantly increased lactate production, indicating enhanced glycolytic flux, while the phosphorylation-resistant mutant showed reduced lactate production, suggesting decreased glycolysis (Fig. 7A).

To further characterize redox alterations induced by PHB2 phosphorylation, NAD^+^/NADH ratios were measured in isolated mitochondria from HepG2 cells expressing PHB2-WT, PHB2–Y34E/Y77E, or PHB2–Y34F/Y77F. The phosphomimetic Y34E/Y77E mutant exhibited the lowest ratio, whereas the phosphorylation-resistant Y34F/Y77F mutant showed the highest compared to WT, consistent with impaired and restored oxidative metabolism, respectively (Fig. 7B). Furthermore, NADP^+^/NADPH ratios were elevated in Y34E/Y77E cells relative to WT in both whole-cell and mitochondrial fractions (Fig. 7C and Supplemental Fig. 5C), suggesting enhanced NADPH consumption to support antioxidant and biosynthetic demands in the phosphomimetic mutant.

Consistent with these redox changes, OROBOROS respirometry revealed that the Y34E/Y77E mutant impaired basal and maximal respiration in intact cells, while the Y34F/Y77F mutant enhanced it (Fig. 7D). Notably, no significant differences in cardiolipin content were observed across these cell lines, indicating that the respiratory defects arise from disrupted PHB2-cardiolipin interactions rather than changes in total cardiolipin levels (Supplementary Fig. 5D and E).

This metabolic reprogramming was evidenced by an inverse relationship between lactate production and oxygen consumption, underscoring that PHB2 phosphorylation shifts cellular metabolism from oxidative phosphorylation toward glycolysis. These alterations were accompanied by changes in proliferative capacity, as EdU incorporation assays demonstrated increased proliferation in Y34E/Y77E-expressing cells and decreased proliferation in Y34F/Y77F-expressing cells (Fig. 7E and F).

The impact of PHB2 phosphorylation on tumor growth was validated in vivo using a xenograft model. Nude mice injected subcutaneously with HepG2 cells expressing different PHB2 variants showed dramatic differences in tumor development, with the phosphomimetic PHB2–Y34E/Y77E mutant accelerating growth and the phosphorylation-resistant PHB2–Y34F/Y77F mutant suppressing it (Fig. 7G and H). Cardiolipin content was significantly reduced in xenograft tumors compared to paired adjacent normal liver tissues, consistent with prior reports, but showed no differences among the three PHB2 variant groups (Supplementary Fig. S5F and G) [36,37]. These in vivo results align with *in vitro* findings and indicate that PHB2 phosphorylation disrupts PHB2-cardiolipin interactions without altering total cardiolipin abundance.

## 4 Discussion

Our study establishes Src-mediated PHB2 phosphorylation as a molecular switch that disrupts the PHB2-cardiolipin-cristae axis, driving metabolic reprogramming in HCC. Phosphorylation at Y34 and Y77 abolishes PHB2-cardiolipin interactions, destabilizing the PHB1/2 supercomplex and triggering OMA1-dependent cristae collapse. This impairs electron transport chain function and decreases the NAD^+^/NADH ratio. In phosphomimetic (Y34E/Y77E) cells, we additionally observed elevated NADP^+^/NADPH ratios in both whole cells and isolated mitochondria, suggesting increased NADPH consumption to support antioxidant defense and biosynthetic demands.

The PHB-cardiolipin interaction represents a critical checkpoint in mitochondrial homeostasis. Cardiolipin is essential for respiratory supercomplex assembly and is highly susceptible to oxidative modification. Previous studies have demonstrated that PHB directly interacts with cardiolipin and that this interaction is important for maintaining cardiolipin content and protecting mitochondrial function under oxidative stress [[38], [39], [40]]. Based on these findings and our observation that Src-mediated PHB2 phosphorylation disrupts the PHB2-cardiolipin interaction (without altering total cardiolipin abundance in cells or xenograft tumors), we speculate that loss of this interaction may render cardiolipin more vulnerable to oxidative damage, although direct measurement of cardiolipin oxidation would be required to confirm this hypothesis.

Our molecular dynamics simulations reveal that PHB1/2 complexes function as cardiolipin organizers, creating specialized membrane microdomains essential for cristae architecture. This finding aligns with evidence that cardiolipin is concentrated at cristae junctions rather than uniformly distributed [13]. The ability of PHB rings to induce local membrane invagination through cardiolipin clustering provides mechanistic insight into cristae morphogenesis. However, cristae morphology is multifactorial and critically depends on additional parameters, including the proton-motive force across the inner membrane (particularly the ΔpH component), respiratory chain supercomplex assembly, MICOS complex integrity, and long-form OPA1 oligomerization [13,[41], [42], [43]]. In this context, Src-mediated dissociation of phosphorylated PHB2 from cardiolipin likely acts cooperatively with impaired respiratory chain activity and reduced ΔpH (evidenced by decreased Complex I- and Complex II-dependent respiration and NAD^+^/NADH ratio) to destabilize cristae architecture in HCC cells.

The Src kinase itself exhibits redox-sensitive activation. In the HCC tumor microenvironment, this creates a potential feed-forward cycle: oxidative stress activates Src, Src phosphorylates PHB2, PHB2 phosphorylation disrupts cristae and impairs respiratory chain function, and respiratory chain dysfunction further exacerbates oxidative imbalance. This cycle may explain the progressive nature of mitochondrial dysfunction in HCC. Our findings integrate the frequent activation of Src in HCC and the correlation between cristae disruption and poor prognosis through the PHB2 phosphorylation mechanism. Furthermore, the observation that OMA1 activation follows PHB complex disruption provides a mechanistic link between cristae structure and OPA1 processing.

From a therapeutic perspective, preventing PHB2 phosphorylation (as demonstrated by the Y34F/Y77F mutant) restores mitochondrial function, normalizes redox balance, suppresses glycolysis, and inhibits tumor growth in vivo. This highlights the Src-PHB2 axis as a targetable vulnerability in HCC.

In conclusion, we have identified Src-mediated PHB2 phosphorylation as a redox-sensitive molecular switch that disrupts the PHB2-cardiolipin-cristae axis, driving metabolic reprogramming in HCC. This mechanism connects oxidative stress, mitochondrial structural remodeling, and metabolic adaptation through a phosphorylation-dependent pathway, providing a molecular framework for understanding HCC biology and identifying therapeutic vulnerabilities. The integration of redox signaling with mitochondrial structural dynamics revealed by this study highlights the importance of considering both biochemical and architectural dimensions of mitochondrial dysfunction in cancer.

## CRediT authorship contribution statement

**Zhehua Shao:** Data curation, Formal analysis, Funding acquisition, Validation, Visualization, Writing – original draft. **Xinnuo Yang:** Data curation, Investigation, Validation. **Binben Wang:** Data curation, Validation. **Xuwen Wang:** Investigation, Validation. **Duoduo Zhao:** Investigation, Validation. **Bingchen Liu:** Funding acquisition, Supervision, Writing – review & editing. **Jinliang Nan:** Conceptualization, Funding acquisition, Supervision, Writing – review & editing.

## Declaration of competing interest

The authors declare that they have no known competing financial interests or personal relationships that could have appeared to influence the work reported in this paper.

## Data Availability

Data will be made available on request.
