# Supplementary material for: Src-mediated PHB2 phosphorylation disrupts mitochondrial cristae through cardiolipin dissociation in hepatocellular carcinoma
Source: Redox Biol. 2026 Feb 7;91:104073. doi: 10.1016/j.redox.2026.104073 (PMC12914208; doi:10.1016/j.redox.2026.104073)
Supplement: Multimedia component 1 [file mmc1.pdf]

Src-mediated PHB2 phosphorylation disrupts mitochondrial cristae through cardiolipin dissociation in hepatocellular carcinoma

Zhehua Shao[a,1,*], Xinnuo Yang[b,1], Binben Wang[c], Xuwen Wang[d], Duoduo Zhao[e], Bingchen Liu [b,f,g,h,*], Jinliang Nan[b,f,g,h,*]

[a]Key Laboratory of Respiratory Disease of Zhejiang Province, Department of Respiratory and Critical Care Medicine, Second Affiliated Hospital of Zhejiang University School of Medicine, Hangzhou, Zhejiang, China.

[b]Department of Cardiology of The Second Affiliated Hospital, School of Medicine, Zhejiang University, Hangzhou, China.

[c]School of Mathematical Sciences, Zhejiang University, Hangzhou, China.

[d]College of Information Science and Electronic Engineering, Zhejiang University, Hangzhou, China.

[e]Bone Marrow Transplantation Center of The First Affiliated Hospital, Zhejiang University School of Medicine, Hangzhou, China.

[f]State Key Laboratory of Transvascular Implantation Devices, Hangzhou, China.

[g]Cardiovascular Key Laboratory of Zhejiang Province, Hangzhou, China.

[h]Research Center for Life Science and Human Health, Binjiang Institute of Zhejiang University, Hangzhou, China.

[1]These authors contributed equally to this work.

[*]Correspondence author. Second Affiliated Hospital of Zhejiang University School of Medicine, Hangzhou, 310009, China

E-mail addresses: jinliangnan@zju.edu.cn (J. Nan); liubingchen111@zju.edu.cn (B. Liu); shaozhehua@zju.edu.cn (Z. Shao).

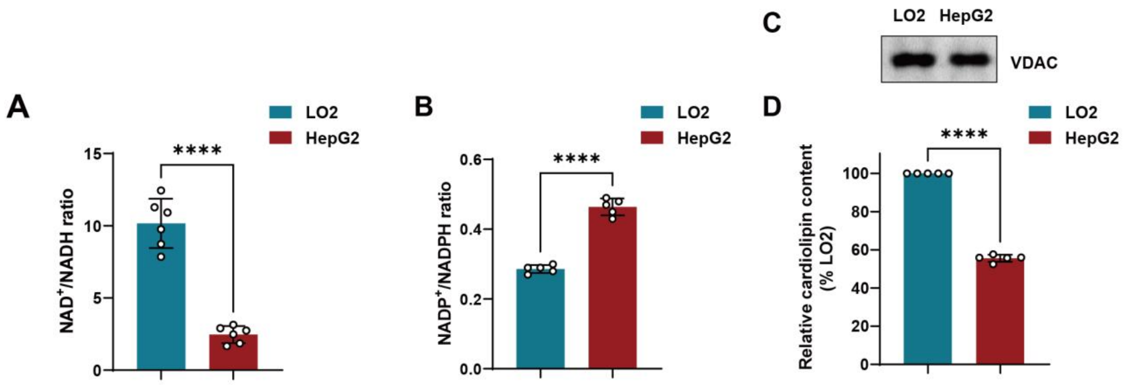

Supplementary Fig. 1 Comparative redox and mitochondrial profiles in LO2 and HepG2 Cells. **A** NAD^+/NADH ratios in LO2 and HepG2 cells measured by enzymatic spectrophotometric method (n=6). **B** $NADP^+$/NADPH ratio comparison between LO2 and HepG2 cells lines (n=5). **C** Western blot results of VDAC expression in LO2 and HepG2 cell lines. **D** Cardiolipin levels in LO2 and HepG2 cell lines quantified by using the NAO fluorescence assay (n=3). All statistical data represent mean ± SD, analyzed using unpaired Student's t-test (A and B). ****$P < 0.0001$.

Src-mediated PHB2 phosphorylation disrupts mitochondrial cristae through cardiolipin dissociation in hepatocellular carcinoma

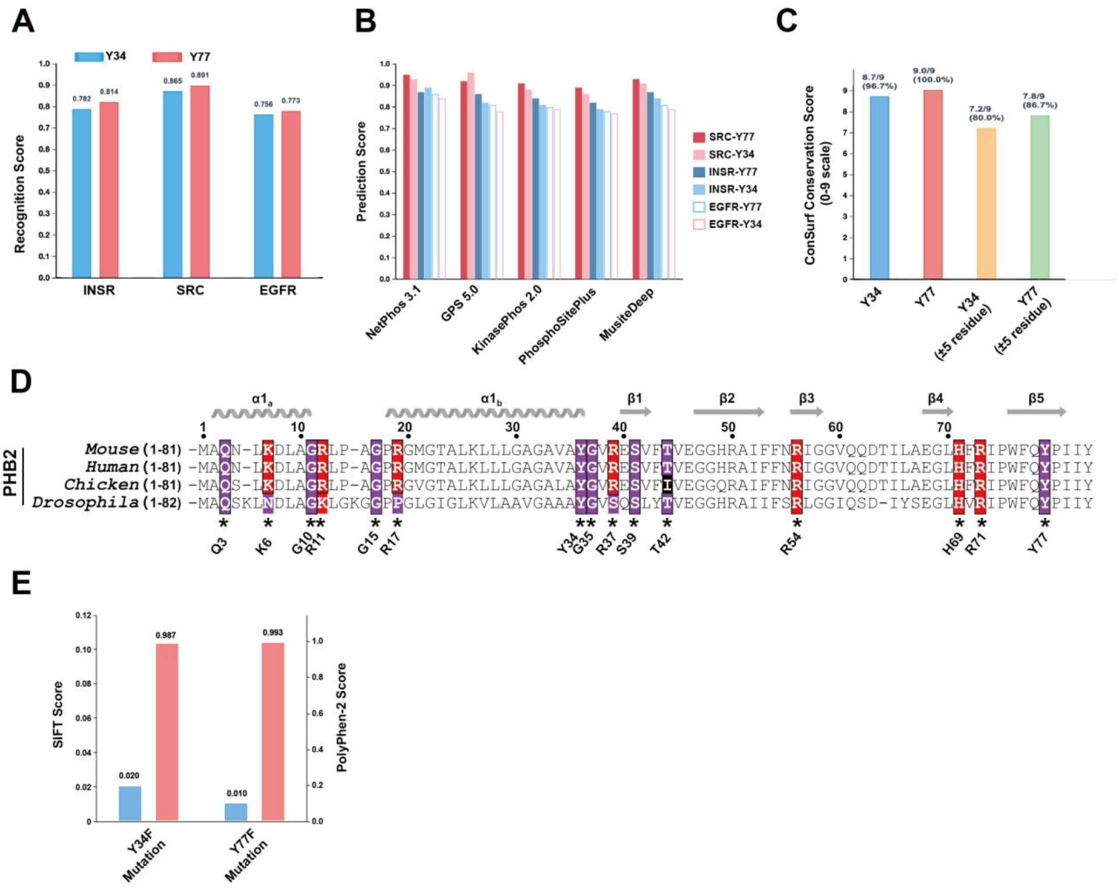

Supplementary Fig. 2 Bioinformatics prediction and analysis of PHB2 phosphorylation sites. **A** Kinase recognition motif analysis using Scansite 4.0 algorithm. Recognition scores for Y34 and Y77 phosphorylation sites by INSR, SRC, and EGFR kinases were calculated based on position-specific scoring matrices (PSSM). Scores normalized to 0-1 range, where >0.75 indicates high confidence recognition and >0.85 indicates very high confidence. **B** Integrated phosphorylation site prediction analysis using multiple algorithms. Prediction scores for PHB2 phosphorylation sites by different kinases (SRC, INSR, EGFR) were calculated using five independent algorithms: NetPhos 3.1 (neural network-based), GPS 5.0 (group-based prediction system), KinasePhos 2.0 (SVM-based), PhosphoBlast (knowledge-based), and MusiteDeep (deep learning-based). Scores were normalized to 0-1 range using min-max standardization. **C** Evolutionary conservation analysis of PHB2 phosphorylation sites using ConSurf algorithm. Conservation scores (1-9 scale, where 9 = completely conserved) were calculated based on multiple sequence alignment of 100 PHB2 homologs from vertebrate

species using MUSCLE algorithm and maximum likelihood phylogenetic method. Shown are scores for Y34, Y77, and combined mutations (LS = less conserved). **D** Analysis of conservation for positively charged and polar amino acids at the N-terminus of PHB2. **E** Functional impact prediction of PHB2 mutations using SIFT and PolyPhen-2 algorithms. SIFT scores range from 0 to 1, where scores <0.05 indicate deleterious mutations. PolyPhen-2 scores range from 0 to 1, where scores >0.85 indicate probably damaging mutations. Both algorithms predict Y34F and Y77F mutations significantly affect PHB2 protein function based on sequence homology and structural features.

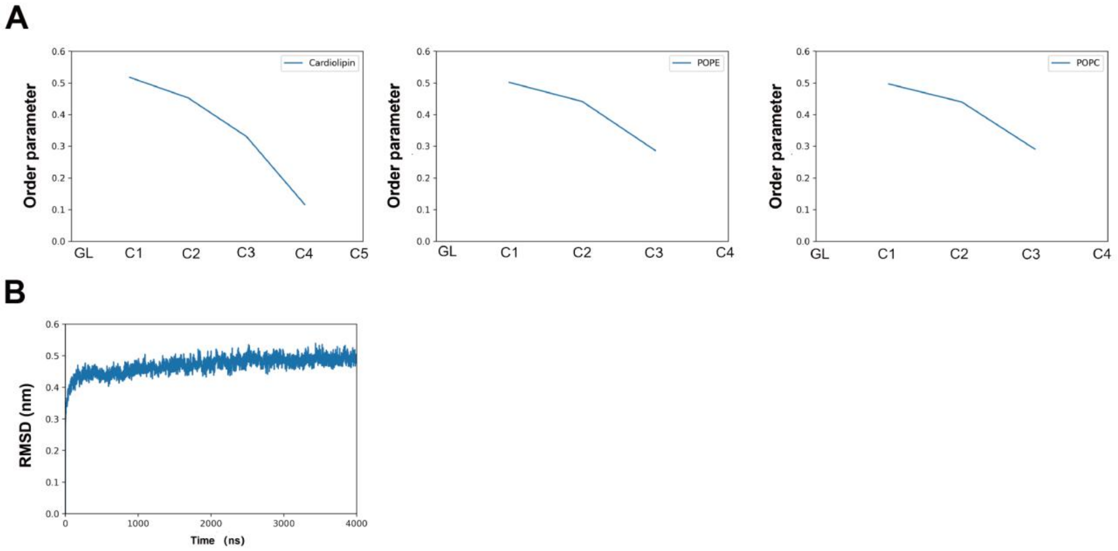

Supplementary Fig. 3 Melting of lipid chains and stability of PHB1/2 during simulations. **A** The order parameters were calculated by taking the average of the four chain order parameter values for cardiolipin, and the average of the two chain order parameter values for POPC and POPE. The constructed lipid bilayer models were evaluated by calculating the atomic order parameter for the lipids. For cardiolipin, POPC, and POPE, the order parameter gradually decreases from the head to the tail of the lipids. **B** Stability of simulated PHB1/2. The trajectory was aligned using the main chain atoms of the initial conformation as a reference, and RMSD was calculated to evaluate its structural stability. After about 100 ns, the structure quickly stabilized, with changes less than 1 Å in value.

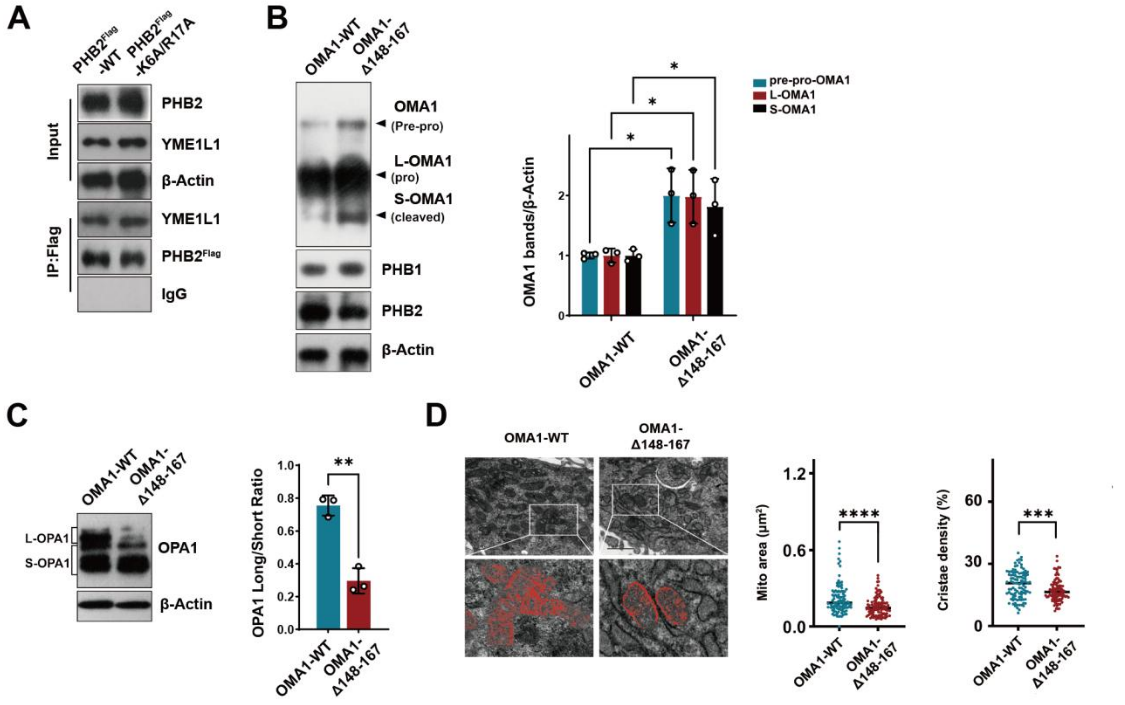

Supplementary Fig. S4 OMA1-cardiolipin interaction and its functional consequences. **A** Co-IP analysis of YME1L1 interaction with PHB2. HEK293T-PHB2-/- cells expressing PHB2-WT or PHB2-K6A/R17A were immunoprecipitated with anti-Flag antibody and probed for YME1L1 and PHB2. B-C Effect of OMA1 cardiolipin-binding domain deletion. **B** Western blot analysis of OMA1 isoforms in HEK293T-OMA1-KD cells expressing OMA1-WT or OMA1-Δ148-167 (lacking cardiolipin-binding domain) (n=3). **C** OPA1 processing showing altered L-OPA1/S-OPA1 ratios with quantification (n=3). **D** TEM analysis of cristae structure with OMA1 mutants. Representative images and quantification showing cristae disruption in cells expressing OMA1-Δ148-167 compared to OMA1-WT. n=100 mitochondria per group, pooled from three independent experiments. Scale bars: 1 μm. All statistical data represent mean ± SD, analyzed using unpaired Student's t-test. *P < 0.05, **P < 0.01, ***P < 0.001, ****P < 0.0001.

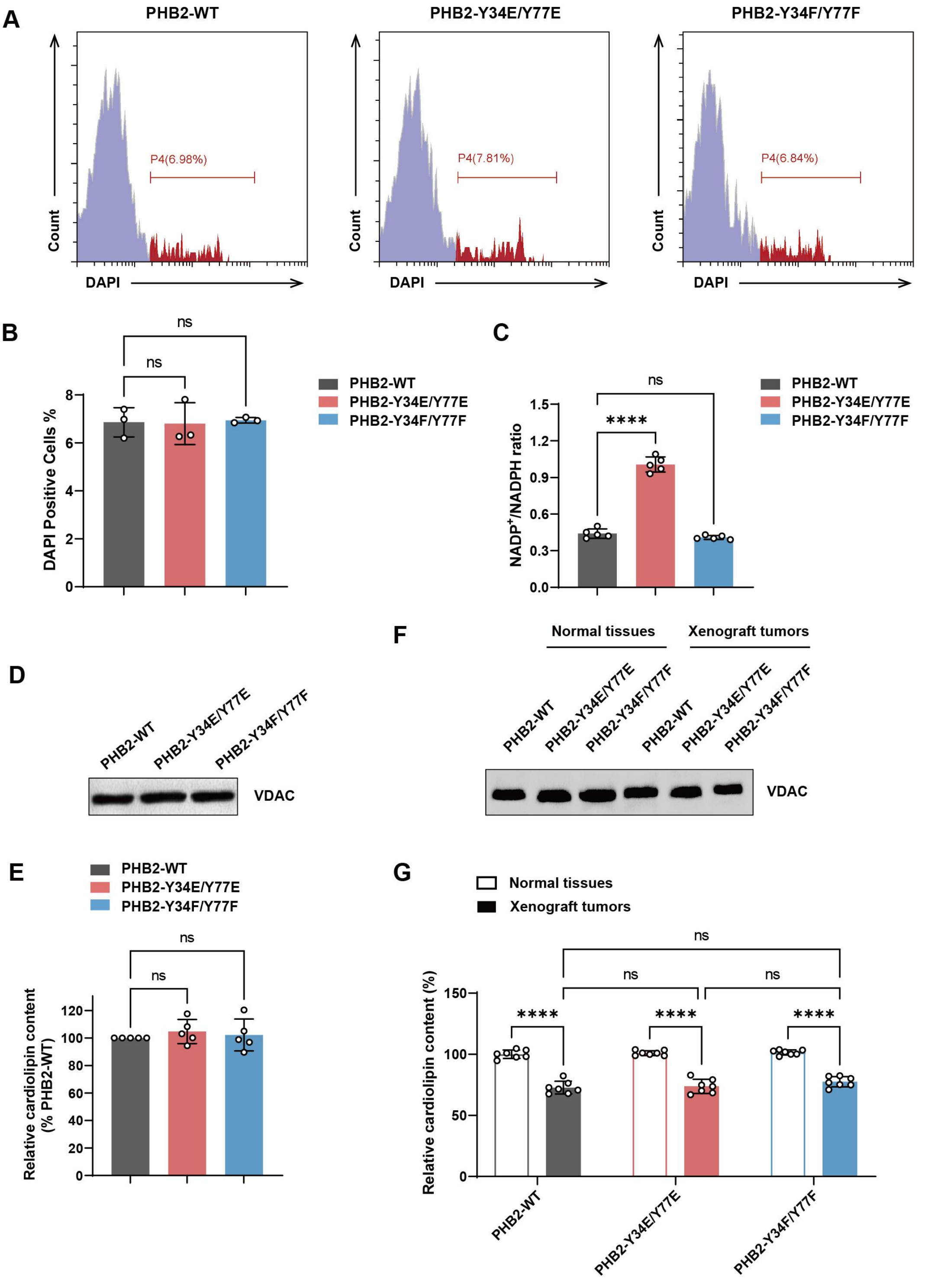

Supplementary Fig. 5 Cell Viability, $NADP^+$/NADPH Ratios, and cardiolipin content in PHB2 variant-expressing HepG2 Cells and xenograft Tumors. **A,B** DAPI-stained cells analyzed by flow cytometry (**A**) and qualified analysis (**B**). **C** $NADP^+$/NADPH ratio in HepG2 cells expressing PHB2-WT, PHB2-Y34E/Y77E, or PHB2-Y34F/Y77F. **D** Western blot results of VDAC

expression in HepG2 cells expressing PHB2-WT, PHB2-Y34E/Y77E, or PHB2-Y34F/Y77F. **E** Cardiolipin levels in HepG2 cells expressing PHB2-WT, PHB2-Y34E/Y77E, or PHB2-Y34F/Y77F, quantified by using the NAO fluorescence assay (n=3). **F** Western blot results of VDAC expression in xenograft tumors derived from HepG2 cells stably expressing PHB2-WT, PHB2-Y34E/Y77E, or PHB2-Y34F/Y77F, as well as in paired adjacent normal liver tissues from the same tumor-bearing mice. **G** Cardiolipin content in xenograft tumors and paired adjacent normal liver tissues. Cardiolipin levels were quantified in tumors derived from HepG2 cells stably expressing PHB2-WT, PHB2-Y34E/Y77E, or PHB2-Y34F/Y77F, as well as in paired adjacent normal liver tissues from the same tumor-bearing mice, using the NAO fluorescence assay. Cardiolipin content was normalized to that of normal liver tissue from the PHB2-WT group (set as 100%) (n=7 mices per group). All statistical data represent mean ± SD, analyzed using one-way ANOVA (B, C, E), and two-way ANOVA (G). ****$P < 0.0001$, ns, not significant.
